# Hypoxia-Inducible Factor 1α (HIF-1α) Counteracts the Acute Death of Cells Transplanted into the Injured Spinal Cord

**DOI:** 10.1523/ENEURO.0092-19.2019

**Published:** 2020-05-11

**Authors:** Brian T. David, Jessica J. Curtin, David C. Goldberg, Kerri Scorpio, Veena Kandaswamy, Caitlin E. Hill

**Affiliations:** 1 Burke Neurological Institute, White Plains, NY 10605; 2 Weill Cornell Medicine, Feil Family Braind and Mind Research Institute, New York, NY 10065

**Keywords:** cell survival, *in vivo* imaging, Schwann cells, spinal cord injury, transcription factor, transplant

## Abstract

Cellular transplantation is in clinical testing for a number of central nervous system disorders, including spinal cord injury (SCI). One challenge is acute transplanted cell death. To prevent this death, there is a need to both establish when the death occurs and develop approaches to mitigate its effects. Here, using luciferase (luc) and green fluorescent protein (GFP) expressing Schwann cell (SC) transplants in the contused thoracic rat spinal cord 7 d postinjury, we establish via *in vivo* bioluminescent (IVIS) imaging and stereology that cell death occurs prior to 2–3 d postimplantation. We then test an alternative approach to the current paradigm of enhancing transplant survival by including multiple factors along with the cells. To stimulate multiple cellular adaptive pathways concurrently, we activate the hypoxia-inducible factor 1α (HIF-1α) transcriptional pathway. Retroviral expression of VP16-HIF-1α in SCs increased HIF-α by 5.9-fold and its target genes implicated in oxygen transport and delivery (VEGF, 2.2-fold) and cellular metabolism (enolase, 1.7-fold). In cell death assays *in vitro*, HIF-1α protected cells from H_2_O_2_-induced oxidative damage. It also provided some protection against camptothecin-induced DNA damage, but not thapsigargin-induced endoplasmic reticulum stress or tunicamycin-induced unfolded protein response. Following transplantation, VP16-HIF-1α increased SC survival by 34.3%. The increase in cell survival was detectable by stereology, but not by *in vivo* luciferase or *ex vivo* GFP IVIS imaging. The results support the hypothesis that activating adaptive cellular pathways enhances transplant survival and identifies an alternative pro-survival approach that, with optimization, could be amenable to clinical translation.

## Significance Statement

To maximize the benefits of cellular transplants for human therapeutic use, there is a critical need to develop strategies that effectively promote transplant survival and permit rapid assessment of transplant survival. The current study (1) identifies the narrow time window in which transplanted cells die within the injured rat spinal cord, thus establishing the time window in which cytoprotection should be targeted to counteract transplanted cell death; (2) tests the effects of elevating HIF-1α on spinal cord transplant survival, thus demonstrating that activating adaptive transcriptional pathways is protective in SCI; and (3) demonstrates, by comparing three approaches to quantifying transplant survival, that until faster and more sensitive methods can be developed, stereology remains the most reliable method.

## Introduction

The death of transplanted cells is a common feature of cell transplants. In the central nervous system, the majority of cells die soon after transplantation (Emgård et al., 2003; Bakshi et al., 2005; Hill et al., 2006, 2007). This undesirable consequence of transplantation, separate from immune-mediated rejection, poses a challenge to the therapeutic use of cellular transplants for neurologic repair. Development of approaches that counteract transplant death are needed to mitigate the deleterious effects of the acute cell death and maximize the clinical utility of cell transplantation.

A necessary first step in developing interventions to counteract transplanted cell death is to accurately establish when post-transplantation (post-TP) the death occurs. In experimental models of spinal cord injury (SCI), 1–35% of cells remain after one week (Barakat et al., 2005; Karimi-Abdolrezaee et al., 2006; Hill et al., 2007), indicating that most transplant death occurs in the first week post-TP. Based on assessments of cell death markers, transplanted cell death peaks within 24 h (Hill et al., 2007). However, the exact time window of transplanted cell death remains to be established. This is due, in part, to the time-consuming nature of histologic quantification of transplanted cells and the fact that few methods currently exist to rapidly screen transplanted cell survival. Establishment of the time frame in which transplanted cells die is necessary to temporally target cell survival interventions. *In vivo* imaging of luminescence can detect expression of reporters (Ratan et al., 2008), antibodies (Aminova et al., 2008), and transplanted cells (Okada et al., 2005; Chen et al., 2006; Kim et al., 2006; Roet et al., 2012), including a reduction in cells over time (Okada et al., 2005; Roet et al., 2012). In the current study, we use *in vivo* bioluminescence imaging to establish the time window of transplanted cell death following engraftment into the injured rat spinal cord. We also test the efficacy of both *in vivo* luminescence imaging and *ex vivo* fluorescence imaging as alternatives to the use of stereology for assessment of transplant survival.

To counteract the potentially deleterious effects of acute transplanted cell death, interventions that promote transplant survival and are amenable to clinical translation are needed. Historically, transplant survival approaches have focused on targeting single factors (Nakao et al., 1994; Mundt-Petersen et al., 2000; Karlsson et al., 2002; Hill et al., 2010). To date, the presence of multiple potential cell death inducers (e.g., hypoxia, oxidative stress, excitotoxicity, lack of substrate/adhesion/growth factors) and the complex cross-talk between cell death pathways has limited the efficacy of this approach. An alternative approach that has proven efficacious, and which does not require identifying the factors responsible for the acute cell death, is the activation of survival pathways. In the injured spinal cord, inclusion of growth factors (Lu et al., 2012; Robinson and Lu, 2017) or enhancement of growth factor signaling (Golden et al., 2007) is effective. In other cell transplantation models, mildly stressing the cells to pre-condition them before engraftment is effective (Murry et al., 1986; Theus et al., 2008; Yu et al., 2013). Although beneficial in preclinical models, implementation as part of a clinical-grade product may prove challenging. Both growth factor signaling and preconditioning activate pro-survival transcriptional programs. Directly engaging transcription factors could provide an alternative means to engage survival pathways.

Among the transcription factors implicated in adaptive responses to cellular stress are members of the hypoxia-inducible factor (HIF) family. The HIFs are DNA-binding transcription factors that consist of an oxygen-labile α-subunit and a constitutively expressed β-subunit. By altering the cell’s metabolic program and gene expression, HIF-α signaling allows cells to sense and adapt to environmental stressors such as decreased oxygen and nutrients. HIFs regulate the expression of genes involved in adaptive transcriptional responses to improve cell survival by enhancing intracellular ATP and oxygen levels and decreasing the production of reactive oxygen species (ROS) in times of hypoxic stress (Semenza, 2007). The genes regulated by HIF-α include key pathways needed for cell survival, such as: glucose metabolism/ATP production, oxygen transport and delivery, and cell growth and fate (Schödel et al., 2013). The diversity of genes regulated by HIF-α shows that its protective effects are not limited to hypoxic environments. In a variety of injury models, elevation of HIF-1α in transplanted cells enhances transplanted cell survival (Theus et al., 2008; Wu et al., 2010). Moreover, pharmacological manipulators of HIFs exist, which makes clinical targeting of this pathway viable.

In this study, to examine the effects of HIF-1α on transplant survival, Schwann cells (SCs) were used. In preclinical SCI models, extensive examination has established that they promote axonal growth and remyelinate axons. A comprehensive review of SC preclinical studies exists, comparing their effects with other cellular therapies (Tetzlaff et al., 2011). Furthermore, a completed phase 1 clinical trial established their safety in humans with SCI (Anderson et al., 2017).

The current study establishes the time window post-TP in which cells die following engraftment into the subacutely injured spinal cord. It tests the hypothesis that transplanted cells die early after implantation due to inadequate activation of transcription factor pathways necessary for cells to adapt to stress and survive. Using viral expression of a non-hydryoxylatable version of HIF-1α, the effect of HIF-1α on cytoprotection is assessed *in vitro* and *in vivo* following transplantation into the injured spinal cord. In an effort to identify methods to facilitate the screening of transplant survival, this study also compares three different techniques for assessment of transplant survival in rats: histologic quantification, *ex vivo* fluorescent imaging of transplanted spinal cords, and *in vivo* bioluminescent imaging.

## Materials and Methods

### Generation of SCs

SCs were isolated from the sciatic nerves of adult female Fischer 344 rats (11–12 weeks of age; Harlan barrier 217; Envigo) following published protocols (Morrissey et al., 1991). The following modification was made: instead of initially growing the nerves in D-10 [DMEM (Thermo Fisher Scientific) with 10% heat-inactivated fetal bovine serum (FBS; HyClone)] on dishes to allow the fibroblasts to grow out, the nerves were allowed to float in D-10+3M [DMEM, 10% FBS, 2 μM forskolin (Sigma-Aldrich), 3.5 μM heregulin (GenWay Biotech), 20 μg/ml pituitary extract (Alfa Aesar), and 0.1% gentamycin (Thermo Fisher Scientific)]. After 10 d, the cells were then dissociated with enzymes [12.5 U/ml dispase (Thermo Fisher Scientific) and 0.5% collagenase (Worthington Biochemicals)], plated, and allowed to grow to confluency before Thy1-complement treating to remove Thy1^+^ fibroblasts. Subsequently, the passage one (P_1_) cells were expanded by plating onto fresh poly-L-lysine (PLL; Sigma-Aldrich) coated 10-cm dishes or frozen for later use.

### Viral manipulation of SCs

Green fluorescent protein (GFP) and luciferase (luc) were used to identify the transplanted cells. SCs were transfected with lentiviruses (LVs). LVs were produced by the Miami Project Viral Vector Core. Viruses were serially applied to the cells at the beginning of each passage. Cells were exposed to the viruses for 18–20 h. LV containing enhanced GFP (LV-GFP; MOI 23) was applied to P_1_ cells and resulted in 94.5% of SCs expressing GFP. LV containing luciferase (LV-luc; MOI 40) was added to P_2_ cells already expressing GFP. A variety of LV-luc constructs were generated to detect light and examine HIF expression in SCs. High levels of luciferase activity were needed to generate enough light to penetrate through the muscle and skin above the transplant site. As a result, cells with the greatest luciferase expression were used. These cells expressed a control construct for examining HIF stabilization (ODD-luc-AYIA; Smirnova et al., 2010). To overexpress HIF-1α in SCs, P_3_ SCs expressing GFP and luc were transfected with a retrovirus expressing HIF in which HIF transcriptional activity is enhanced by the inclusion of the VP16 transactivation domain (TAD; RV-VP16-HIF; MOI 10) or a control virus (VP16; MOI 10; Kung et al., 2000; Aminova et al., 2005). The viruses were previously generated by transfection of 293T cells with pBAB-puro-HIF-1α-VP16 or a control plasmid (pBABE-puro-VP16; Harvard Gene Therapy Initiative) and kindly provided by Dr. Rajiv Ratan. The RV-VP16-HIF virus results in the expression of a fusion protein encoding amino acids (aa) 1–529 of HIF-1α and 78 aa of the VP16 TAD. The VP16 TAD is a potent TAD located within the carboxyl terminus of herpes simplex virus type 1 transcription factor VP16. When the VP16 TAD is fused to a transcription factor, it amplifies its activity. By creating a fusion protein with aa 1–529 of HIF-1α that contains the DNA binding domains of HIF-1α, but lacking the oxygen degradation domain, this virus produces a HIF-1α fusion protein with both enhanced stability and transcriptional activity. Retroviruses were added in the presence of polybrene (4 μg/ml). Cells were subsequently expanded and frozen. For experiments, SCs between P_4_ and P_8_ were used.

### RNA isolation and PCR

RNA was collected from SCs by adding TRI reagent (Zymo Research) to the SCs and then isolating the RNA using the Direct-zol RNA Miniprep (Zymo Research) according to the manufacturer’s instructions. To confirm viral expression of HIF, RNA was isolated. RNA was quantified using the Nanodrop spectrophotometer (Thermo Fisher Scientific). cDNA was generated using Superscript III First Stand Synthesis System (Thermo Fisher Scientific) and PCR was subsequently performed using primers for HIF-1α, VP16 and β-actin (as per Aminova et al., 2005) and PCR products identified following electrophoresis.

### Protein isolation and western blotting

Nuclear and cytoplasmic proteins were isolated from cultured SCs using the NE-PER kit (Thermo Fisher Scientific), as per the manufacturer’s instructions. Cells were collected in CER I buffer with protease inhibitors by mechanically scraping. During isolation of the nuclear fraction, NaCl (200 mM; Sigma-Aldrich) was added at 10 min to facilitate release of bound nuclear proteins and 1 μl (≥250 U) of benzonase nuclease (Sigma-Aldrich) was added at 20 min to digest nucleotides. Protein concentration was determined by DC assay, as per the manufacturer’s instructions (Bio-Rad). For western blotting, the protein samples were quantified and either used immediately or stored at –80°C. Protein samples were heat-denatured (95°C, 5 min), mixed with 6x loading buffer with SDS (Boston BioProducts), and loaded onto a 4–15% gradient precast SDS-PAGE gel (Bio-Rad). Using electrophoresis, proteins were separated (120 V, 1.5 h, RT, running buffer; Boston BioProducts), and subsequently transferred to a nitrocellulose membrane (100 V, 1.5 h, 4°C, transfer buffer; Boston BioProducts) with 20% methanol. Nuclear protein (50 μg), was assessed for HIF-1α (1:1000, Novus NB 100-105) and HIF-2α (1:500, Novus NB 100-122). Nuclear protein (20 μg) was assessed for VP16 (1:1000, ab4808, Abcam). Cytoplasmic protein (20 μg) was assessed for VEGF (1:500, NB100-2381, Novus Biologicals) and enolase (1:1000, NB100-65252, Novus Biologicals). β-Actin (1:10,000, Sigma-Aldrich) or GFP (1:1000, G6539, Sigma-Aldrich) was used as a loading control. Antibodies were diluted in Odyssey blocking buffer (LI-COR). Primary antibodies were incubated overnight at 4°C. LI-COR secondary antibodies (1:10,000; IRDye 800CW, IRDye 680LT) were incubated for 90 min at room temperature. Membranes were imaged and band intensities were quantified using the Odyssey Infrared Imaging System (LI-COR). For each antibody, a minimum of three separate samples per condition were run and analyzed from the same blot. For some experiments, SCs were treated for 24 h with 200 μM deferoxamine (Sigma-Aldrich), a known HIF stabilizer. Samples from these cells were used as a positive control for localization of HIF proteins on HIF western blots.

### *In vitro* cell death assays

To assess the effects of HIF on SC survival *in vitro,* LV-GFP-luc-VP16 HIF SCs (VP16-HIF SCs) or LV-GFP-luc SCs (control) were plated onto 96-well plates and grown for 41–47 h. Medium was then removed, and fresh D-10+3M was added along with various inducers of cell death. Hydrogen peroxide (H_2_O_2_; 1–1000 μM, Sigma-Aldrich) was added to dishes in which 10,000 SCs were plated to induce oxidative damage. SC survival was assessed 3 h later. To determine the impact of other activators of cell death, 25,000 cells per well were plated and SC survival was assessed 24 h later. Tunicamycin (0–8 μM, Sigma-Aldrich) was added to activate the unfolded protein response. Thapsigargin (0–6 μM, Sigma-Aldrich) was added to induce calcium release from intracellular stores. Camptothecin (0–12 μM, Sigma-Aldrich) was added to induce DNA damage. SC survival was assessed by MTS assay (Promega). Phenazine methosulfate (PMS) was added to tetrazolium compound: 3-(4,5-dimethylthiazol-2-yl)-5-(3-carboxymethoxyphenyl)-2-(4-sulfophenyl)-2H-tetrazolium, inner salt (MTS) immediately before addition to the cells, as per the manufacturer’s instructions. The medium was replaced by MTS solution either 3 h (H_2_O_2_ assays) or 24 h (all other assays) after treatment. Cells were incubated with MTS at 37°C, 6% CO_2_ for 4 h before absorbance was read using the SpectraMax i3 cytometer/spectrophotometer (Molecular Devices) at 490 nm. Percentage of cell survival was calculated for each condition per plate relative to untreated cells of the same type (VP16-HIF or control SCs). Briefly, the percentage of cells surviving in each well was calculated [(well absorbance – blank absorbance)/average absorbance of untreated cells]. Next, for each treatment for a given cell type on a given plate, the mean for the technical replicates was determined to establish the percentage survival for each condition (i.e., each independent sample). Finally, percentage of cell survival per condition was calculated by averaging the results from each independent sample. Results represent the means of three to four independent assays in which the technical replicates (three to four/replicate) were averaged. For all assays, 2 μl of lysis solution (9% weight/volume Triton X-100; Thermo Fisher Scientific) was added to assess maximal death.

### SCI and transplantation

Female Fischer 344 rats (Harlan, barrier 217, 9–11 weeks of age) were anesthetized with isoflurane (2–3%). To expose the spinal cord, a laminectomy was performed to remove the dorsal process of thoracic vertebrae 9 (T9). The lateral processes of T8 and T10 were clamped and a 200-kdyn injury was induced using the Infinite Horizon impactor (Precision Systems and Instrumentation). The impact curve was checked for hit quality at the time of injury and saved. The injury site was inspected for a bruise before suturing the muscles closed in two separate anatomic layers. The skin was closed with wound clips. The temperature of the rats was monitored and maintained throughout the surgery using a thermoregulated heating pad. Buprenorphine (0.05 mg/kg) was given twice a day for the first 2 d postsurgery to alleviate pain. Lactated Ringer’s (10 ml) was given one to two times per day for the first 2 d postsurgery to prevent dehydration. Gentamycin (5 mg/kg) was given once a day for the first 7 d postsurgery to prevent infections. Along with food and water ad libitum, wet food pellets were provided to help maintain the rat’s weight. Rats were housed in pairs, or in groups of three, and cages were placed on thermoregulated heating pads (half on-half off) to assist with thermoregulation for the first week postsurgery.

Seven days after SCI, rats underwent a second surgery to transplant the cells. Rats were anesthetized with isoflurane (2–3%) or ketamine and xylazine (80 mg/kg:10 mg/kg). The anesthetic used was kept consistent for each experiment. The previous incision site was reopened and the laminectomy site re-exposed. The dorsal vertebral process of T8 was clamped to stabilize the rat and 6 μl of cells was injected into the injury site. Cells were injected using a pulled glass capillary tube with silicone plug attached to a Hamilton syringe affixed to a nanosyringe pump (KD Scientific) attached to a stereotactic device. Cells were injected at a rate of 1 μl/min at a depth of 1–1.25 mm. The capillary was left in place for an additional 3 min to allow the pressure to equilibrate before removal. The laminectomy site was then sutured closed in two anatomic layers and the skin was closed with wound clips. Surgical and postsurgical care (e.g., temperature monitoring, drugs) was administered as after the initial SCI surgery. All animal procedures were performed in accordance with the Weill Cornell Medicine animal care committee’s regulations.

### Optimization of *in vivo* bioluminescent imaging (IVIS)

To examine transplant survival in rats over time, and to assess whether IVIS imaging could be used to measure transplant survival more rapidly than stereological quantification, changes in light emission over the first 7 d following transplantation were quantified using the IVIS 100 (PerkinElmer). Initial experiments examined the sensitivity of the IVIS for detecting different numbers of transplanted cells and compared intraperitoneal injection of D-luciferin with intravenous injection following transplantation of 2 × 10^6^ GFP-luc SCs. Intravenous administration via the tail vein resulted in a more rapid increase in light production than intraperitoneal administration (data not shown). The time course of the decrease in light emitted from the transplants over time was compared to the number of transplanted cells at 3 d (*n* = 4) and 7 d (*n* = 5). A subset of these rats were quantified by stereology to determine the number of surviving SCs at 3 d (*n* = 3) and 7 d (*n* = 4). Spinal cords from two of the rats that underwent IVIS imaging were not available for histologic analysis.

### Transplantation of HIF-1α SCs and assessments of transplant survival

To assess whether increasing HIF-1α in SCs promoted transplanted SC survival, the effect of overexpressing HIF was tested in a fully-blinded, randomized, *in vivo* SCI experiment. Both the treatment group assignment and the order of the cell types transplanted were assigned randomly before cell collection and transplantation. Twenty-six rats underwent a T9, 200 kdyn IH SCI (one rat died post-SCI). The 25 remaining rats received SC transplants 7 d post-SCI and were euthanized at 7 d post-TP (14 dpi). Rats received transplants of 2 × 10^6^ SCs directly into the injury epicenter. The three transplant treatment groups were: GFP-luc SCs (control SCs, *n* = 9), GFP-luc-VP16-SCs (VP16 SCs, *n* = 8), GFP-luc-VP16-HIF-1α SCs (VP16-HIF SCs, *n* = 8). Using up to three different methods, transplant survival was assessed in the same rats. Following transplantation, 12 of the 25 rats (*n* = 4/group) underwent *in vivo* bioluminescent imaging using the IVIS Spectrum (PerkinElmer) to detect the transplanted cells based on their luciferase expression. *In vivo* bioluminescent imaging was performed on these 12 rats daily for the first 3 d post-TP, the time window in which we determined that transplanted cell death occurs. Seven days post-TP, after perfusion and isolation of the spinal cords (*n* = 25), *ex vivo* GFP fluorescent imaging of the spinal cords was performed using the IVIS Spectrum. The spinal cords were then sectioned with a cryostat and the number of surviving SCs was quantified by Stereo Investigator (MBF Bioscience). By sequentially assessing the same spinal cords, we were able to compare non-biased stereology, which reliably detects differences in transplant survival, with alternative methods of transplant survival quantification. In this study, we compared the results of the stereological quantification of surviving SCs with *ex vivo* fluorescent imaging of light emission from GFP and *in vivo* bioluminescent imaging of luciferase activity within a single set of experimental rats. On histologic inspection of the 25 spinal cords, 21 rats were determined to have received good transplants (control SCs *n* = 7; VP16 SCs *n* = 6; VP16-HIF SCs *n* = 8) and were included in the stereological and *ex vivo* imaging analyses. For *in vivo* bioluminescence, all imaged rats that were determined to have received good transplants were included in the analysis (control SCs *n* = 3; VP16 SCs *n* = 4; VP16-HIF SCs *n* = 4).

Exclusion of rats from the analysis was based on assessment of the quality of the transplant, which was undertaken before unblinding of the experiment. The transplants were assessed histologically for tissue section completeness and location of the transplanted cells in both the rostral-caudal and dorsal-ventral axes relative to the lesion epicenter. The stereological results were then compared with the transplant surgery notes and notes on the histologic assessment of the transplants. Two rats with small transplants were excluded because of problems with the injection that were noted at the time of transplantation. Two additional rats were excluded because the transplant was not located within the lesion epicenter.

### IVIS luminescent imaging

IVIS luminescent imaging was performed using either the IVIS 100 or IVIS Spectrum. Rats were anaesthetized with isoflurane (1–2%; Henry Schein). Baseline images were acquired before intravenous injection of D-luciferin (150 mg/kg, Gold Biotechnology) into the tail vein. IVIS imaging was performed daily as close as possible to the time of transplantation each day. Following administration of D-luciferin, IVIS images were acquired by collecting the amount of light emitted over 5 min (exposure: 5 min; binning: medium; F/stop: 1; field of view: 400 cm^2^; emission filter: open). Rats were imaged continuously in 5-min intervals for up to 60 min to determine the time of maximum light emission. The image with maximum light was used to quantify the amount of light emitted. A region of interest (ROI) of 6.41 cm^2^ was centered over the site of maximum light and both the average radiance (photons/s/cm^2^/steradian) and the total flux (photons/s) were determined. Because total flux is derived from average radiance, results from both showed a similar profile. Average radiance is presented. In cases where light was not initially detected within 0–30 min, rats received a second injection of D-luciferin and were re-imaged. If, following a second injection, light was not detected, the data were excluded from analysis for that time point only.

### Tissue collection

Three days (*n* = 3) or 7 d (total, *n* = 29; IVIS assessment of timing of TP death, *n* = 4; assessment of VP16-HIF SC survival, *n* = 25) after transplantation, rats were sacrificed by lethal injection of ketamine (Henry Schein)/xylazine (Henry Schein). Spinal cords were collected after transcardiac perfusion of heparinized (Henry Schein) 0.9% saline followed by 4% paraformaldehyde (Sigma-Aldrich). Paraformaldehyde-fixed tissue was postfixed overnight before transferring to 30% sucrose-PBS (Sigma-Aldrich) to facilitate cryopreservation for histologic analysis.

### *Ex vivo* IVIS fluorescent imaging

The amount of fluorescent light emitted from the GFP transplants was quantified in the isolated fixed spinal cords using *ex vivo* imaging with the IVIS Spectrum. Spinal cords were placed on a Petri-dish with a black backing and inserted into the IVIS Spectrum. The number of photons emitted by the GFP^+^ SCs within the spinal cord was determined using the following IVIS settings: excitation/emission = 500/540; exposure = 0.5 s; binning = 4, F-stop = 2; field of view = B. A photographic image of the spinal cord overlaid with the photon intensity count was generated. The ROI of GFP fluorescence was then auto-sized and the average radiant efficiency [(p/s/cm^2^/sr)/(μW/cm^2^)] determined for each case. To account for variability in light intensity between images, the same settings were used to show visible photons on all the images (min = 3.25 × 10^8^; max = 10 × 10^9^).

### Histology and quantification of SC survival and transplant volume

A 15-mm block of the spinal cord containing the injury epicenter was cut into longitudinal sections (20 μm) on the cryostat. Four sets of serial sections were collected onto charged slides. Nuclei were labeled by incubating tissue sections with 1:1000 Hoechst (Sigma-Aldrich) in PBS (pH 7.4) for 1 h. Slides were air dried and coverslipped with Vectashield (Vector Laboratories) before imaging. To quantify the total number of transplanted GFP^+^ SCs and the GFP^+^ transplant volume, Stereo Investigator was used. The transplant was outlined at 10× magnification. Using the Cavalieri function, transplant volume was quantified at 10× magnification (grid size was 200 × 200 μm). Using the optical fractionator function, the number of GFP^+^ cells were quantified at 63× magnification (grid box size was 150 × 400 μm and the sampling box was 50 × 50 μm).

### Imaging

Tissue was examined, imaged, and quantified using either a Zeiss Axiovert 200M or a Zeiss AxioImager M2 equipped with Stereo Investigator. Virtual tissue sections were acquired at 10× magnification. Similar Exposure and Gain settings were maintained for all image acquisition for a given experiment. Confocal images were acquired using a Zeiss LSM 510 META confocal microscope.

### Chemical and biosafety

All viral work was performed using biosafety level 2 procedures and approved by the Weill Cornell Medicine Institutional Biosafety Committee. Individuals working with viruses and chemicals received institutional biosafety and chemical training and donned appropriate personal protective equipment when executing experiments with hazardous material.

### Statistics

All statistics were performed using SPSS (version 22; IBM). An overview of statistical tests used can be found in Table 1. Specifics of the statistical tests used are included in the results.

**Table 1.** Statistical table

	Data structure	Type of test	Power
a	Normal distribution	Univariate ANOVA, repeated contrasts	0.89
b	Normal distribution	t-test	0.08
c	Normal distribution	Univariate ANOVA	1.00
d	Normal distribution	Univariate ANOVA	0.14
e	Normal distribution	Univariate ANOVA	1.00
f	Normal distribution	Univariate ANOVA	1.00
g	Normal distribution	Univariate ANOVA	1.00
h	Normal distribution	Univariate ANOVA	0.94
i	Normal distribution	Univariate ANOVA	1.00
j	Normal distribution	Univariate ANOVA	0.95
k	Normal distribution	Univariate ANOVA	1.00
l	Normal distribution	Univariate ANOVA	0.22
m	Normal distribution	Univariate ANOVA	1.00
n	Normal distribution	Univariate ANOVA	0.85
o	Normal distribution	Univariate ANOVA	0.76
p	Normal distribution	Univariate ANOVA	0.43
q	Normal distribution	Univariate ANOVA, repeated contrasts	0.89
r	Normal distribution	Univariate ANOVA	0.43
s	Normal distribution	General linear model	0.53

## Results

### Transplanted cell number does not change between 2 and 7 d post-TP

**Figure 1.**
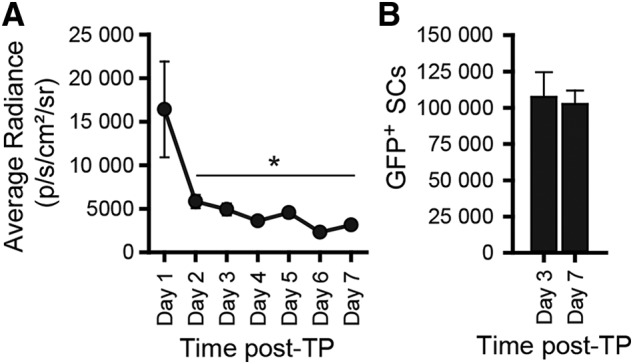
Decrease in transplanted cells occurs within 2 d post-TP, after which time there is no further decrease in cell number; 2 × 10^6^ GFP-luc SCs were transplanted into the injured spinal cord 7 d postinjury. Transplanted cell survival was assessed in live rats by *in vivo* bioluminescent imaging (***A***). The amount of light produced by luciferase activity was quantified daily in the IVIS following D-luciferin administration for up to 7 d (days 1–3, *n* = 8–9; days 4–7, *n* = 3–5). Following tissue collection, the number of surviving GFP^+^ transplanted cells was quantified at 3 d (*n* = 3) or 7 d (*n* = 4) post-TP in the fixed, sectioned spinal cords by stereology (***B***). Mean ± SEM; *p = 0.004. *Figure Contributions*: Veena Kandaswamy and Kerri Scorpio performed the experiment. Caitlin Hill analyzed the data.

Transplanted cells die early after transplantation into the injured spinal cord (Barakat et al., 2005; Hill et al., 2006, 2007; Karimi-Abdolrezaee et al., 2006). However, the exact time course of transplanted cell death remains to be established. To counteract transplanted cell death, it is necessary to establish the window in which the death occurs. Several studies have shown that the amount of light detected using bioluminescent imaging correlates with cell number transplanted and that the light decreases following the transplantation of cells (Okada et al., 2005; Takahashi et al., 2011; Roet et al., 2012; Nishimura et al., 2013). Using the IVIS imaging system, bioluminescent imaging was performed for either 7 d (*n* = 4) or 3 d (*n* = 3) to establish when post-TP transplanted cells die. Following intravenous injection of D-luciferin via the tail vein, it took 5–10 min for the amount of detected light to reach the maximal level, as measured by average radiance. The maximal amount of light produced by the luciferase-expressing SCs decreased significantly over time post-TP (univariate ANOVA: *F*_(6,36)_ = 3.302, *p* = 0.011^a^). The amount of light detected at day 2 was significantly lower than on day 1 (K matrix: 1 d vs 2 d, *p* = 0.004^a^). After day 2, no further reduction in light production was detected. The amount of light detected was similar from 2 to 7 d post-TP (K matrix: all other contrast *p* > 0.05^a^; days 2–7: Ryan–Einot–Gabriel–Welsch homogenous subset, *p* = 0.98^a^; Fig. 1*A*). Thus, based on the amount of light detected by *in vivo* bioluminescent imaging, we established that the number of transplanted cells decreases within the first 48 h post-TP.

To confirm that the number of surviving transplanted cells did not differ after the first few days, when the light levels in the IVIS had plateaued, the spinal cords from the IVIS imaged rats were examined histologically. The number of GFP^+^ SCs within the transplants were quantified at 3 d (*n* = 3) and 7 d (*n* = 4) post-TP. Stereological quantification confirmed that the number of surviving transplanted SCs did not differ between 3 d and 7 d post-TP (t-test, df: 5, *p* = 0.8^b^; Fig. 1*B*). This fits with previous work in which necrosis and apoptosis of transplanted SCs was detected at 24 h but not 3 d post-TP (Hill et al., 2007). These experiments led us to conclude that the death of the cells occurs within a narrow time window immediately post-TP and concludes within 2–3 d.

Multiple factors are postulated to contribute to transplanted cell death (e.g., cell processing, transplant procedure, injury/transplant environment, transplant rejection). Examination of the factors that contribute to transplanted cell death indicate that the injury/transplant environment is the likely culprit (Hill et al., 2006, 2007, 2010). Cell processing and the transplant procedure account for <10% of the observed cell death (Hill et al., 2007). In a model of transplant rejection, immunosuppression does not block this early death (Hill et al., 2006). Transplant survival is enhanced by delaying engraftment for at least 7 d (McDonald et al., 1999; Hill et al., 2006), but is not further augmented by waiting for the chronic injury site to develop (Barakat et al., 2005). This suggests that the environment generated by the cell transplant is a key contributor to acute transplanted cell death, and likely why cell death is a common feature of all cell transplants.

### Expression of VP16-HIF-1α in SCs increases HIF-1α, but not HIF-2α, and increases protein expression of HIF target genes

Although the exact cause of this death has not been established, the complexity and redundancy of the pathways involved in tissue damage after SCI support targeting coordinated responses instead of a single gene, protein, or pathway to improve transplant survival. Activation of transcriptional programs leads to alterations in targeted downstream cassettes of genes that act in concert to alleviate the relevant stress(es) and their by-products. Harnessing the power of cellular transcriptional programs could abrogate the need to identify and target individual cell death inducers.

HIF-1α is a member of a family of transcription factors that regulate transcriptional responses involved in cellular metabolism and wound healing (Ruthenborg et al., 2014; Yang et al., 2014; Pugh, 2016). HIF target genes are involved in cell growth and fate, mitochondrial functioning, glycolysis and glucose metabolism, and oxygen transport and delivery (Semenza, 2012), all of which could benefit transplanted cell survival. In the current series of experiments, we sought to elevate HIF-1α in SCs and test whether it could promote the survival of the transplanted cells.

**Figure 2.**
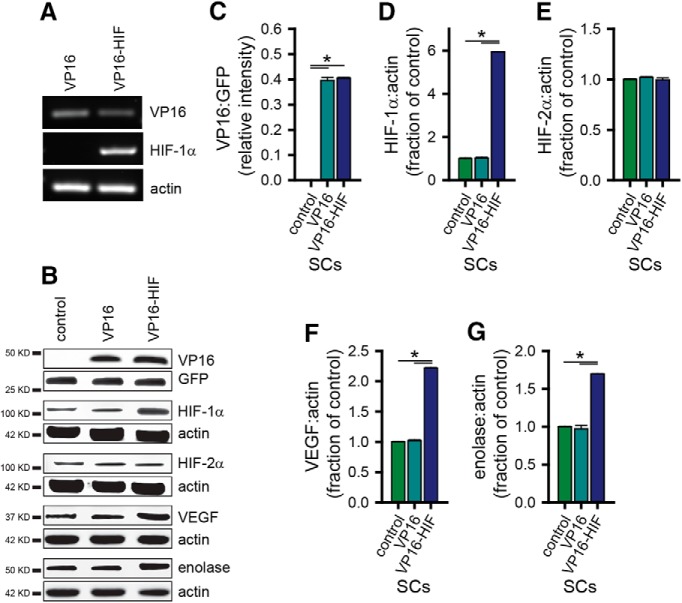
HIF-1α protein and HIF-1α target genes are elevated in SCs following retrovirus administration. Expression of mRNA for VP16, HIF-1α, and β-actin in transfected SCs was confirmed by PCR in VP16 and VP16-HIF SCs (***A***). Protein expression of VP16, HIF-1α, HIF-2α, VEGF, enolase, and protein loading controls (GFP or β-actin) in transfected SCs was assessed by western blotting (***B***). Protein expression was normalized to the loading control. Relative intensity of VP16 expression is shown in ***C***, and the fold change in protein expression relative to control SCs is shown for HIF-1α (***D***) HIF-2α (***E***), VEGF (***F***), and enolase (***G***). Values are as follows: VP16 (control: 0.0 ± 0.0; VP16: 0.40 ± 0.01; VP16-HIF: 0.41 ± 0.00); HIF-1α (control: 1.0 ± 0.04; VP16: 1.0 ± 0.04; VP16-HIF: 5.9 ± 0.01); HIF-2α (control: 1.0 ± 0.01; VP16: 1.0 ± 0.01; VP16-HIF: 1.0 ± 0.02); VEGF (control: 1.0 ± 0.01; VP16: 1.02 ± 0.02; VP16-HIF: 2.2 ± 0.01); enolase (control: 1.0 ± 0.01; VP16: 1.0 ± 0.04; VP16-HIF: 1.7 ± 0.00); *n* = 3/group. Mean ± SEM, *p < 0.0001. *Figure Contributions*: Veena Kandaswamy generated the VP16-HIF cells. Veena Kandaswamy performed the PCRs. Ying Dai performed the western blottings. Ying Dai and Caitlin Hill analyzed the data.

Experimentally, HIF-1α levels can be manipulated genetically by the generation of non-hydroxylatable HIF-1α (Kung et al., 2000; Aminova et al., 2005). Using a retrovirus to express a VP16-HIF-1α fusion protein that contains a transcriptionally-active, non-degradable form of HIF-1α (Kung et al., 2000; Aminova et al., 2005), we elevated HIF-1α levels in SCs to physiologic levels. Following transduction of SCs with retroviruses expressing either VP16-HIF-1α or VP16, VP16 mRNA (Fig. 2*A*) and protein (Fig. 2*B*,*C*) were detected in both VP16 and VP16-HIF SCs. Human HIF-1α mRNA (Fig. 2*A*) was detected in VP16-HIF-1α SCs but not VP16 SCs. At the protein level, HIF-1α (Fig. 2*B*,*D*), but not the closely-related HIF-2α (Fig. 2*B*,*E*), was significantly elevated in VP16-HIF-1α SCs (HIF-1α: ANOVA: *F*_(6,2)_ = 7993.5, *p* < 0.0001^c^, Bonferroni *post hoc*, *p* < 0.0001; HIF-2 α: ANOVA: *F*_(6,2)_ = 0.878, *p* = 0.463^d^), thus demonstrating the specificity of the virus for HIF-1α. Compared to control SCs, HIF-1α levels were increased by 5.9 ± 0.2-fold in VP16-HIF SCs. This is within the physiologic increase in HIF-1α achieved with hypoxia treatment in other cell types [e.g., stem cells: Theus et al., 2008; Wakai et al., 2016; bone marrow stromal cells (BMSCs): Theus et al., 2008], where HIF-1α increases by 2- to 6.5-fold. It is, however, less than the 8–14-fold increase achievable following pharmacological treatment of SCs with the HIF stabilizer, deferoxamine (unpublished data). Expression of VP16-HIF in SCs resulted in the elevation of two representative HIF target genes: VEGF, a protein important in tissue vascularization, and enolase, a glycolytic enzyme. VEGF increased 2.2 ± 0.2-fold (ANOVA: *F*_(6,2)_ = 3223.6, *p* < 0.0001^e^, Bonferroni *post hoc*, *p* < 0.0001; Fig. 2*B*,*F*). Enolase increased 1.7 ± 0.01-fold (ANOVA: *F*_(6,2)_ = 277.1, *p* < 0.0001^f^, Bonferroni *post hoc*, *p* < 0.0001; Fig. 2*B*,*G*). Thus, retroviral expression of a non-degradable form of HIF-1α fused to the VP16 TAD elevated HIF-1α levels in SCs to the physiologic levels, and was sufficient to elevate the expression of HIF’s transcriptional targets. The level of expression, although within the physiologic range, was submaximal.

### Elevation of HIF in SCs via retroviral expression of VP16-HIF enhances SC survival in response to oxidative stress but not ER stress or DNA damage

The transcriptome regulated by HIF contains a core set of genes activated in response to hypoxia (Benita et al., 2009), along with cell-specific transcriptional changes (Chi et al., 2006). This generates both specificity and diversity in the transcriptional response (Lendahl et al., 2009). Activation of HIF adaptive signaling is primarily associated with cytoprotection, as evidenced by its protective effect on transplanted cells when elevated either directly or indirectly by induction with hypoxia or pharmacological preconditioning (Chu et al., 2008; Theus et al., 2008; Wu et al., 2010). However, among HIF’s target genes are BNIP3 and NIX, which are associated with mitochondrial damage, apoptosis and autophagy (Ney, 2015). Moreover, in a neuronal cell line *in vitro*, HIF expression augments glutamate-induced oxidative stress-mediated cell death (Aminova et al., 2005). Thus, at least in some contexts, elevation of HIF-1α can lead to enhancement of cell death.

**Figure 3.**
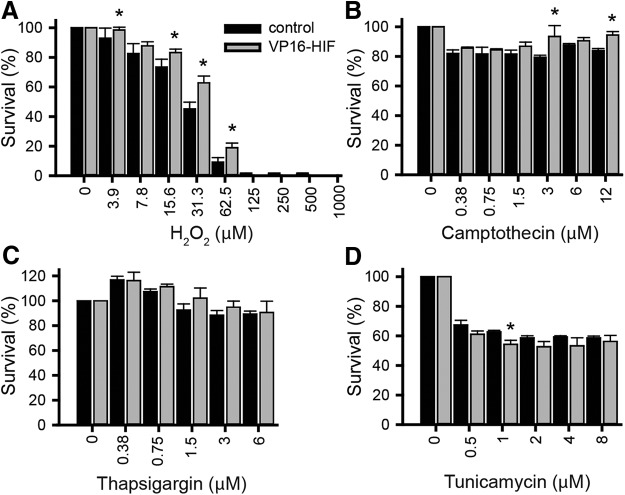
Elevation of HIF-1α inhibits, enhances, or has no effect on SC survival in response to different cell death inducers. SC cultures in 96-well plates were treated with various doses of H_2_O_2_ (***A***), camptothecin (***B***), thapsigargin (***C***), or tunicamycin (***D***) and survival assessed at either 3 h (H_2_O_2_) or 24 h (other inducers) by MTS assay to assess the effects of elevation of HIF in response to oxidative stress, DNA damage, ER Ca^++^ release, and the UPR, respectively; *n* = 3 independent experiments/condition. Mean ± SEM. *, ***A***, 3.9 μM, *p* = 0.03; 16.3 μM, *p* = 0.006; 31.3 μM, *p* < 0.0001; 62.5 μM, *p* = 0.03; ***B***, 3 μM, *p* = 0.002; 12 μM, *p* = 0.014; ***D***, 1 μM, *p* = 0.037. *Figure Contributions*: Caitlin Hill and Jessica Curtin performed the experiments. Caitlin Hill analyzed the data.

To determine whether expression of HIF-1α was cytoprotective for SCs against oxidative stress-induced cell death, the effects of overexpression of HIF-1α on cell survival was assessed *in vitro* by MTS assay. To model oxidative damage *in vitro*, SCs were exposed to H_2_O_2_ (0–1000 μM) for 3 h. This dose range resulted in a dose-dependent reduction in SC survival from 0 to 150 μM H_2_O_2_ (ANOVA: *F*_(9,40)_ = 246.1, *p* < 0.0001^g^; K matrix: 0–150 μM, *p* < 0.05). There were significantly more VP16-HIF SCs compared to control SCs across a range of H_2_O_2_ concentrations (3.9, 15.6, 31.3, and 62.5 μM; Fig. 3*A*; ANOVA: *F*_(1,40)_ = 12.9, *p* = 0.001^h^, *post hoc*: Fisher’s LSD, *p* ≤ 0.05). The extent by which HIF-1α protected the SCs varied with H_2_O_2_ concentration. At the LD50 dose of H_2_O_2_ (31.5 μM), 45.2 ± 4.58% of control SCs survived, whereas 62.8 ± 4.64% of VP16-HIF SCs survived. Expression of HIF-1α increased SC survival by 39%. This is equivalent to the protection achieved by calpain inhibition (Hill et al., 2010). Therefore, expression of HIF-1α in SCs was sufficient to protect SCs against oxidative stress-mediated cell death. These results were expected, given HIF-1α’s established role in mitigating ROS-mediated damage (for review, see Thomas and Ashcroft, 2019), but were contrary to the results found in a neuronal cell line (Aminova et al., 2005).

Under some contexts, HIF expression can be deleterious (Aminova et al., 2005). The effects are both cell type (Vangeison et al., 2008) and injury inducer-dependent (Aminova et al., 2005). As the inducers of transplanted cell death remain to be identified, we further examined the survival of SCs by evaluating their survival *in vitro*. Several additional known inducers of cellular toxicity, including: DNA damage, ER stress, and protein unfolding were examined to establish the context in which HIF-1α leads to SC cytoprotection.

To assess the ability of HIF to protect SCs against DNA damage, SCs were exposed to camptothecin (0–12 μM) *in vitro* for 24 h (Fig. 3*B*). Camptothecin significantly reduced the viability of SCs across the range of concentrations tested (ANOVA: *F*_(6,28)_ = 8.5, *p* < 0.0001^i^). However, despite using a range of concentrations effective in other cells *in vitro* (Aminova et al., 2005), most of the SCs (80–87%) remained viable. Only 0.375 μM camptothecin treatment differed from the preceding concentration (K matrix: 0 vs 0.375 μM, *p* < 0.0001, all other contrast *p* > 0.05; 0.375–12 μM). The lack of a dose-response curve across a range of concentrations that are toxic for HT22 cells indicates that SCs are relatively resistant to DNA damage-induced death via camptothecin. Although the effect of camptothecin on SC viability was modest, HIF expression protected SCs against camptothecin-mediated cell death (ANOVA: *F*_(1,28)_ = 13.7, *p* = 0.001^j^). Viability of VP16-HIF SCs was significantly enhanced in response to application of 3 or 12 μM camptothecin (*post hoc*: Fisher’s LSD, 3 μM, *p* = 0.002, 12 μM, *p* = 0.014). At 3 μM, HIF-1α increased cell survival by 17.7% (79.4 ± 1.38% of control SCs survived compared to 93.5 ± 7.31% of HIF-1α SCs). At 12 μM, HIF-1α increased survival by 12.5% (83.9 ± 1.35% of control SCs survived compared to 94.4 ± 2.34% of HIF-1α SCs). Thus, although SCs are relatively resistant to DNA damage-induced cell death, elevating HIF is still sufficient to afford some protection to SCs against DNA damage-induced death.

ER stress was induced by exposure of SCs to thapsigargin (0–6 μM) for 24 h (Fig. 3*C*), which results in Ca^++^ release from intracellular stores. Thapsigargin significantly altered SC viability across a range of concentrations (ANOVA: *F*_(5,24)_ = 9.26, *p* < 0.0001^k^); however, across concentrations proven toxic for HT22 cells (Aminova et al., 2005), SCs did not show a dose-response curve and an LD50 was not obtained. Thapsigargin decreased cell viability to 88–93% of control at high doses (>1.5 μM). Unexpectedly, at low doses (0.375–0.75 μM), thapsigargin increased cell viability to 107–117% of control (0 μM). Although not tested, this is likely due to altered SC proliferation. Only application of 0.375 and 1.5 μM thapsigargin resulted in changes in viability relative to the preceding concentration (K matrix: 0 vs 0.375 μM, *p* = 0.002; 0.75 vs 1.5 μM, *p* = 0.02). Based on the relatively small decrease in SC viability across a range of thapsigargin concentrations that are toxic to other cells, it appears that SCs were relatively resistant to ER stress induced by thapsigargin. Elevating HIF in SCs did not alter SC viability in response to thapsigargin (ANOVA: *F*_(5,1)_ = 1.5, *p* = 0.225^l^). Larger alterations in SC survival in response to ER stress are needed to establish whether HIF-1α levels impact ER stress-mediated cell death in SCs.

Accumulation of misfolded or unfolded proteins can lead to cell death. To induce protein unfolding in SCs, tunicamycin (0–8 μM) was administered for 24 h (Fig. 3*D*). Administration of tunicamycin induces autophagy, which can either be protective, or, if autophagy is excessively activated or disrupted, lead to cell death (Ding et al., 2007). Tunicamycin administration resulted in a dose-dependent decrease in SC viability (ANOVA: *F*_(5,24)_ = 82.7, *p* < 0.0001^m^), but only at the lowest concentrations tested. There was a significant decrease in viability from the preceding tunicamycin concentration at 0.5 and 1 μM (K matrix: 0.5 vs 0 μM, *p* < 0.005; 1 vs 0.5 μM, *p* = 0.04), Beyond 1 μM, at concentrations that are toxic to HT22 cells (Aminova et al., 2005), 59–63% of SCs continued to survive (Fig. 3*D*), indicating that SCs were relatively resistant to tunicamycin treatment. Expression of HIF-1α in VP16-HIF SCs did not counteract the reduction in SC viability in response to tunicamycin. Rather, VP16-HIF SCs had a small reduction in viability compared to control SCs (ANOVA: *F*_(1,24)_ = 9.7, *p* = 0.005^n^). This significant difference was detected at only a single concentration (*post hoc*: Fisher’s LSD, 1 μM, *p* = 0.04). At 1 μM tunicamycin, HIF expression decreased SC viability by 13.3% (62.7 ± 1.11% of control SCs survived compared to 54.5 ± 2.69% of VP16-HIF SCs). Although a significant decrease in survival was observed when HIF-expressing SCs were treated with tunicamycin, the effect was restricted to a small change at a single dose of the five doses tested. Overall, SCs were relatively resistant to cell death induced by activation of the unfolded protein response. This likely reflects the importance of autophagy in the de-differentiation and reprograming SCs (Gomez-Sanchez et al., 2015).

Previous reports indicate that the effects of HIF-1α on cell survival are influenced by both the cell type and cell death mechanism (Aminova et al., 2005). In SCs, the *in vitro* assays indicate that elevation of HIF via retroviral expression of VP16-HIF-1α results in substantial protection against oxidative stress (38.9% increase). In other models of cell stress, the ability of elevated HIF-1α in SCs to protect SCs was more variable and of a limited magnitude (<20% increase or decrease). In SCs, HIF-1α resulted in modest protection (DNA damage), no alteration (ER stress), or a reduction (unfolded protein response) in survival. These results fit with the overall view that activation of HIF-1α adaptive pathways is cytoprotective, but that its effects are context dependent.

### Elevation of HIF in SCs via retroviral expression of VP16-HIF promotes transplanted SC survival 7 d post-TP

Having established that expression of HIF-1α in SCs led to elevated levels of target genes in adaptive HIF pathways (Fig. 2), and that this protected SCs against oxidative stress-induced cell death *in vitro* (Fig. 3*A*), we tested whether elevating HIF in SCs enhances their survival following transplantation into the injured spinal cord.

Following transplantation of SCs into the injured spinal cord 7 dpi, stereological quantification determined that significantly more SCs survived when VP16-HIF was expressed (number of surviving SCs: control, 128,400 ± 18,900; VP16, 142,200 ± 30,200, and VP16-HIF: 172,400 ± 30,100; ANOVA: *F*_(18,2)_ = 5.259, *p* = 0.016^°^; *post hoc*: one-tail Fisher’s LSD, VP16-HIF vs control, *p* = 0.0025, VP16-HIF vs VP16, *p* = 0.0265). The increase in survival by 34.3% in VP16-HIF SCs compared to control SCs reflects a very large effect size (Hedges’ g = 1.72). The 21.2% increase in survival compared to VP16 SCs reflects a large effect size (Hedges’ g = 1.00). As many studies have assessed transplant size as a measure of transplant survival, we also quantified transplant volume. Transplantation of VP16-HIF SCs was associated with a slight, but significant, increase in transplant volume relative to control SCs, but not VP16 SCs (transplant volume: control, 1.6 ± 0.15 mm^3^, VP16, 1.9 ± 0.29 mm^3^, VP16-HIF, 2.1 ± 0.47 mm^3^; ANOVA: *F*_(18,2)_ = 5.162, *p* = 0.016^p^; *post hoc*: one-tail Fisher’s LSD, VP16-HIF vs control, *p* = 0.0025, VP16-HIF vs VP16, *p* = 0.11). The transplant volumes for all groups were within the size of SC transplants reported previously (Golden et al., 2007; Hill et al., 2010). The improvement of transplant survival achieved with HIF SCs is similar to that observed in some studies in which HIF-1α is targeted in either neural stem cell/progenitor cell or BMSC transplants (Theus et al., 2008; Wang et al., 2018).

HIF is associated with enhanced migration of cancer cells (Araos et al., 2018) and neural crest cells (Barriga et al., 2013). Larger transplants could arise from better transplant survival and/or enhanced transplant migration. No evidence for migration of the SCs out of the transplants and across the SC-astrocyte boundary were detected in this study. The presence of astrocytes and inhibitory molecules within the glial scar is known to prevent SC migration (Afshari et al., 2010).

In this study, we sought to test whether the elevation of HIF-1α and activation of its transcriptional programs could provide an alternative approach to the use of preconditioning or growth factor augmentation for promoting the survival of the transplanted cells. Elevating the expression of HIF-1α by 5.9-fold increased the expression of the HIF target genes VEGF and enolase by 2.2 and 1.7-fold, respectively. This level of HIF activity was associated with a 20–35% increase in transplant survival. Thus, we demonstrate that overexpression of a single transcription factor is sufficient to protect SCs transplanted into the injured spinal cord. Further optimization is needed to obtain the level of protection afforded by HIF-1α in other transplant models either alone (Wu et al., 2010; Wakai et al., 2016) or with the inclusion of growth factors along with cell transplants into the injured spinal cord (Golden et al., 2007; Lu et al., 2012; Robinson and Lu, 2017).

### *Ex vivo* fluorescent imaging of spinal cords and *in vivo* luminescent imaging of rats did not detect differences in transplant survival

A barrier to the development and identification of new strategies that promote cell survival are the methods currently used to assess transplant survival accurately. As part of this study, we assessed whether *ex vivo* fluorescent imaging (Fig. 3*H*,*I*) or *in vivo* bioluminescence imaging (Fig. 3*J*,*K*) could detect a difference in transplant survival. Similar to the earlier experiment, IVIS imaging was sensitive enough to detect a difference in light emission over time (ANOVA: *F*_(8.89,1.111)_ = 11.955, *p* = 0.006^q^, sphericity corrected; *post hoc*: Bonferroni, d1 vs d2, *p* = 0.01, d1 vs d3, *p* = 0.031). Neither IVIS imaging of live animals, nor *ex vivo* imaging of isolated spinal cords, was sensitive enough to detect the increase in transplant survival observed histologically (*ex vivo* IVIS GFP: ANOVA: *F*_(18,2)_ = 2.448, *p* = 0.115^r^; *in vivo* IVIS luciferase: ANOVA: *F*_(8,2)_ = 0.032, *p* = 0.969^s^). Based on power analyses, a minimum of 14–18 rats would be needed to detect a significant difference (power = 0.8, α = 0.05) between the three groups using either luminescence or GFP as an outcome on the IVIS, respectively. Although *ex vivo* and *in vivo* imaging of the transplants is feasible; a large number of samples would be needed to reliably detect differences. Thus, these methods are unlikely to be a more rapid alternative to stereological quantification of transplanted cells.

## Discussion

The results of the current studies establish that transplanted cells die before 2 d post-TP. The narrow window of cell death followed by the stabilization of the number of transplanted cells supports that acute, rather than prolonged, manipulation of survival pathways could be sufficient to counteract transplanted cell death. HIF-1α is involved in cellular adaptations to stress. Here, we demonstrate that the elevation of HIF-1α increases cell survival in both an *in vitro* model of transplanted cell injury and following transplantation of SCs into the subacutely injured spinal cord. We show that stabilization of HIF-1α elevates HIF transcriptional targets and is sufficient to protect SCs against oxidative stress and DNA damage-induced cell death, two major mechanisms of cell death following SCI (Ahuja et al., 2017). When HIF-1α-expressing SCs are transplanted into the injured spinal cord, where multiple inducers of cell death are present (Ahuja et al., 2017), more cells survive. These experiments support the utility of harnessing cellular adaptive responses to protect them from subsequent transplant-induced cellular stress. They also establish that targeting a transcription factor (HIF-1α) and activation of its target pathways is cytoprotective for cells transplanted into the injured spinal cord. This identifies an alternative approach to those currently used which target individual cell death inducers or signaling pathways, or involve the inclusion of multiple growth factors along with the transplanted cells. Importantly, strategies exist to transiently elevate HIF-1α transcriptional programs (e.g., hypoxic preconditioning or pharmacological pretreatment of transplanted cells), which, if effective, will enable the elevation of protective transcriptional programs using a pretreatment approach. Pretreatment of the cells could provide a more clinically-feasible method for enhancing transplant survival than current pro-survival approaches, which require inclusion of multiple proteins along with the cells.

Histologic quantification of transplant survival is laborious. The identification of optimal transplant conditions would greatly benefit from methodological advances that are both sensitive and facilitate reliable quantification of transplanted cell survival. Several new methods exist for quantifying transplant survival, including *in vivo* and *ex vivo* bioluminescence and fluorescence. Having established that HIF-1α increased transplant survival using stereological quantification, we assessed the ability of *ex vivo* fluorescent imaging and *in vivo* bioluminescent imaging to detect the improvement in survival. Neither approach detected the improvement in transplant survival. Thus, for rat spinal cord transplants, histologic quantification using non-biased stereology remains the most sensitive and reliable method for determining differences in transplant survival.

### Transplanted cells die within the first 2–3 d when transplanted into the subacutely injured spinal cord

Several different cell types are currently in human testing for SCI repair (clinicaltrials.gov). Initial reports from human clinical trials indicate that cell transplants are safe (Tabakow et al., 2013; Shin et al., 2015; Anderson et al., 2017) but that the functional effects of cell transplants alone are limited. Ultimately, the effects of cell transplants will depend on both the cell type transplanted and the summation of the changes (both beneficial and potentially detrimental) that occur within the transplanted tissue. Because transplants persist long term, an often-overlooked concern which may impact transplant and tissue function is the acute death of the majority of transplanted cells (Barakat et al., 2005; Hill et al., 2006, 2007; Karimi-Abdolrezaee et al., 2006).

To both maximize transplant efficacy and design superior anti-cell death interventions, establishing when this death occurs is needed. Here, using daily *in vivo* bioluminescence imaging and histologic confirmation, we narrow the time window of transplanted cell death to the first 2 d post-TP (Fig. 1). The current reduction in bioluminescence following SC transplantation parallels both the decrease in transplanted cell number (Barakat et al., 2005; Hill et al., 2007; Pearse et al., 2007) and bioluminescence determined in previous rodent spinal cord transplant experiments (Okada et al., 2005; Kumagai et al., 2009; Takahashi et al., 2011; Ozdemir et al., 2012; Roet et al., 2012; Nishimura et al., 2013; Iwai et al., 2014). Although our bioluminescence results are similar to previous reports, previous studies generally report a slightly more prolonged decrease in bioluminescence, where light levels plateau by 4 dpi (Okada et al., 2005; Ozdemir et al., 2012), 7 dpi (Nishimura et al., 2013), or 15 dpi (Roet et al., 2012) compared to the 2 d observed here. Our results with SC grafts in subacute rat contusion SCIs most resemble those of neural stem cell/progenitor cell grafts in subacute mouse contusion SCIs, where bioluminescent activity decreases by ∼80% and plateaus by 4 d post-TP, the earliest time point presented (Okada et al., 2005; Nishimura et al., 2013). Differences in injury model, cell type, transplant location, mechanism of luciferase expression, and time of transplantation between the studies likely contribute to the variability in the results reported between this and previous studies. Collectively, this and previous studies assessing transplant survival/death establish that transplant death occurs in all cell transplants, and that the death occurs in a narrow, acute window immediately post-TP.

The largest contributor to transplant death is likely the transplant environment (Hill et al., 2006; Nishimura et al., 2013; Piltti et al., 2013). SCI results in complex biochemical and cellular changes that could contribute to the death of the transplanted cells, including: hypoxia, ischemia, oxidative and nitrosative stress, inflammation and immune mediators, decreased growth factors, and an altered extra-cellular matrix (Ahuja et al., 2017). This has led to the use of a multimodal transplant paradigm that includes a substrate, multiple growth factors, and a pharmacological inhibitor of cell death along with the cells (Lu et al., 2012; Robinson and Lu, 2017). Although effective, clinical translation of this approach will be difficult. The acute window of death determined here indicates that if interventions that protect the cells acutely can be identified, long-term inclusion of multiple factors is unlikely to be required.

### HIF-1α protects SCs from oxidative stress and enhances transplant survival

HIF-1α is a key regulator of cellular adaptations to stress, including hypoxic, ischemic and oxidative stress. In the injured brain, elevating HIF-1α either by hypoxic preconditioning or overexpression is sufficient to reduce transplant death (Theus et al., 2008) and enhance transplanted survival (Wu et al., 2010; Wakai et al., 2016). Recently, hypoxic preconditioning was shown to protect BMSC transplants in the injured spinal cord (Wang et al., 2018). HIF-1α regulates the transcription of >100 targets, including key mediators of angiogenesis and tissue vascularization (e.g., VEGF; Forsythe et al., 1996; Ryan et al., 1998) and glycolysis and glucose metabolism (e.g., enolase; Semenza et al., 1994, 1996). Here, we show that elevating HIF-1α in SCs enhances nuclear levels of HIF-1α in the cells and HIF transcriptional targets implicated in angiogenesis (VEGF; Fig. 2) and glycolysis (enolase; Fig. 2).

*In vitro*, HIF-1α’s effects on cell survival depend on the mechanism of cell death induction (Fig. 3*A–D*). This is similar to previous work in neurons (Halterman and Federoff, 1999; Aminova et al., 2005). Although HIF-1α is generally considered protective, among its transcription targets are pro-death genes linked to apoptosis and autophagy (Chen et al., 2009) and, under some circumstances, HIF-1α augments cell death (Aminova et al., 2005; Vangeison et al., 2008). We observed that elevation of HIF-1α protects SCs against oxidative stress (Fig. 3*A*) and DNA damage (Fig. 3*B*), is slightly detrimental on activation of the unfolded protein response (UPS; Fig. 3*D*), and has no effect on ER stress (Fig. 3*C*). With the exception of the response to oxidative stress, the magnitude and breadth of the survival changes in SCs in response to the inducers of cell death tested was limited. This is in contrast to neurons, where HIF-1α affords substantial protection against DNA damage, ER stress, and UPS activation, but is pro-death in response to glutamate-induced oxidative stress (Aminova et al., 2005). It is well-established that cellular sensitivity to cell death and HIF-1α’s effects are cell type and context-dependent (Chen et al., 2009). Compared to neurons (Aminova et al., 2005), SCs were relatively resistant to DNA damage, ER Ca^++^ release and induction of autophagy by initiation of the unfolded protein response. This could reflect a greater adaptability of SCs to harsh environments and/or the ability of SCs to modify their phenotype (e.g., SCs undergo substantial remodeling when they de-differentiate following peripheral nerve injury). This could account for the slightly higher survival rate of SCs (Barakat et al., 2005; Hill et al., 2007; Pearse et al., 2007) than neurons or neural progenitors (Barker et al., 1996; Karimi-Abdolrezaee et al., 2006) following transplantation. It is postulated that mild hypoxia leads to expression of adaptive HIF-1α responses, whereas severe or prolonged exposure leads to the expression of pro-death genes (Halterman et al., 1999). The greater adaptability of SCs to harsh environments could also account for the differences between SCs and neurons exposed to oxidative stress. The specific cell death inducers that impact transplant survival remain to be established. Further studies to identify and test the mediators of transplanted cell survival/death are necessary to establish the context under which HIF-1α and other pro-survival interventions affect transplant survival in a cell and environment-specific manner.

Elevation of HIF-1α in SCs protects SCs against oxidative damage (Fig. 3*A*), to which SCs have been shown to be sensitive (Hill et al., 2010). In response to H_2_O_2_ treatment, HIF-1α protected SCs to a similar level as inhibition of calpain-mediated cell death (Hill et al., 2010). In the vestibular system, SCs elevate HIF in response to oxidative stress, but it is not sufficient to prevent death (Riva et al., 2007), suggesting that the endogenous adaptive response of SCs to oxidative stress is insufficient to counteract the oxidative damage. Similarly, examination of the levels of HIF-1α in the spinal cord following transplantation indicate that SCs fail to initiate the HIF-1α adaptive response within the first 8 h after transplantation (unpublished data). Blocking lipid peroxidation, a consequence of oxidative damage, *in vitro* protects SCs but is insufficient to protect a variety of transplanted cells (Karlsson et al., 2002), including SCs (Hill et al., 2010). It is possible that counteracting oxidative stress is the wrong target for transplant protection. Alternatively, current treatments may not effectively reduce the generation of ROS and subsequent oxidative stress. As HIF-1α is a regulator of diverse biological pathways, including oxygen supply and utilization (Dengler et al., 2014), it may afford better protection against oxidative damage than previous approaches. Moreover, HIF not only activates transcriptional programs that lead to decreased ROS production (Semenza, 2011), but, in response to hypoxia, generation of ROS is necessary to increase HIF-1α, making it a sensor of ROS (Chandel et al., 2000). The mechanism by which HIF is stabilized in response to ROS is not yet known, however, ROS are postulated to increase HIF-1α levels by interfering with HIF’s hydroxylation by the HIF-prolyl hydroxylases at Pro^402^ and Pro^564^ and factor inhibiting HIF (FIH) at asparagine (Asn^803^; Semenza, 2011). This implies that oxidative stress could augment HIF-1α stability and activity, further increasing its cellular functions. In the current study, the HIF construct used contains aa 1–529 of HIF. Thus, one of the hydroxylation sites, Pro^402^, is retained. It is therefore possible that the enhanced protective effects in response to oxidative stress arise from greater elevation in the levels of HIF in this condition.

**Figure 4.**
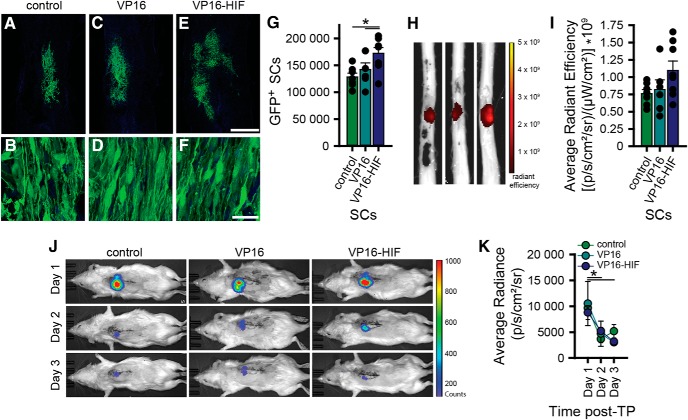
HIF increases the survival of transplanted cells. This is detectable histologically by stereological quantification, but not by *ex vivo* fluorescent imaging of spinal cords or *in vivo* bioluminescent imaging; 2 × 10^6^ GFP-luc SCs were transplanted into the injured spinal cord 7 d post-SCI [control (*n* = 7), VP16 (*n* = 6), or VP16-HIF (*n* = 8)]. Seven days post-TP, transplant survival was quantified by histology (***A–G***) and *ex vivo* fluorescent imaging of spinal cords for GFP (***H***, ***I***). In a subset of the rats [control (*n* = 3), VP16 (*n* = 4), or VP16-HIF (*n* = 4)], transplant survival was assessed by performing daily *in vivo* bioluminescent imaging on days 1–3 post-TP (***J***, ***K***). Representative images of the transplants and the cells within the transplants 7 d post-TP are shown for each group (***A–F***). More SCs survive when they express VP16-HIF, as determined by stereology (***G***). Results of quantification of the cell number by stereology (***G***). Images of representative spinal cord showing radiant efficiency of the *ex vivo* imaging for transplant GFP fluorescence 7 d post-TP (***H***). Quantification of radiant efficiency (***I***). Images of photon counts for bioluminescence activity of the transplanted cells for the first 3 d post-TP for each cell type transplanted (***J***) and quantification of light emitted (average radiance; ***K***). Mean ± SEM; *, ***G***, control versus VP16-HIF, *p* = 0.0025; VP16 versus VP16-HIF, *p* = 0.027; ***K***, d1 versus d2, *p* = 0.01; d1 versus d3, *p* = 0.031. *Figure Contributions*: Caitlin Hill, Brian David, Jessica Curtin, and David Goldberg performed the experiment. Caitlin Hill and Brian David analyzed the data.

HIF-1α increases the survival of SCs transplanted into the subacutely injured spinal cord (Fig. 4*A–G*). This result is similar to previous studies in which direct or indirect elevation of HIF-1α enhances transplant survival of cells transplanted into the damaged brain (Theus et al., 2008; Wu et al., 2010; Wakai et al., 2016), heart (Zhang et al., 2001), and pancreas (Stokes et al., 2013). Although expression of HIF-1α protected the cells, the increase in transplant survival with HIF-1α was smaller than in previous spinal cord transplant studies targeting trophic support, anoikis, and/or cell death signaling (Golden et al., 2007; Hill et al., 2010; Patel et al., 2010; Lu et al., 2012; Robinson and Lu, 2017; Cerqueira et al., 2018). It is well established that prolonged overexpression of HIF in transplanted cells induces tumor formation (Kung et al., 2000). We chose to use low constitutive HIF expression to mitigate this potential problem. A greater increase in HIF-1α transcription may be required over the low-level HIF-1α induction of this study. Higher levels of HIF transcription are achievable with higher viral MOIs and pharmacological stabilization of HIF (unpublished data). Alternatively, activation of other transcription factors that mediate other key cellular programs (e.g., Nrf2 and NFκβ) may provide greater survival benefits than HIF-1α.

Elevation of HIF-1α can result in the doubling of transplant size (Wu et al., 2010; Wakai et al., 2016). This is substantially greater that the enhancement in survival achieved in the current study. Several factors could contribute to this difference in transplant survival including differences in the levels of HIF-1α in the cells post-TP. One limitation of this and previous studies is that although HIF-1α levels were measured in the cells before TP, they have not been measured post-TP. Cell type differences could also affect both the sensitivity of the cells to the transplant environment and the specifics of the transcriptional program(s) activated (Chi et al., 2006; Benita et al., 2009; Lendahl et al., 2009). Previous studies have examined stem cells or BMSCs, cell types that reside in low-oxygen niches. It is currently unclear how oxygen tension impacts adult de-differentiated SCs and which specific transcriptional programs are activated in SCs in response to elevation of HIF-1α.

This study establishes that increasing HIF-1α is cytoprotective for transplanted SCs. It also demonstrates the feasibility of targeting HIF (and other transcription factors) as an approach to enhance transplanted cell survival. It supports the hypothesis that activating adaptive responses in transplanted cells can protect them following transplantation. This paves the way to test pharmacological interventions that temporarily elevate HIF (or other transcription factors) before transplantation to test whether overcoming the initial 2-d window in which the cells die is sufficient. Ongoing studies in the lab are testing the efficacy of clinically-feasible paradigms to elevate HIF-1α transiently and substantially.

### Development of additional, rapid, reliable methods to quantify transplant survival is needed

Stereology proved to be the most sensitive method for detecting differences in transplant survival. In the injured spinal cord, IVIS imaging is able to detect a decrease in transplanted cells over time (Fig. 1*A*; Okada et al., 2005; Takahashi et al., 2011; Roet et al., 2012; Nishimura et al., 2013). In cancer studies, it detects increases in tumor cells (Rehemtulla et al., 2000). Under the current conditions, neither *in vivo* bioluminescence imaging (Fig. 4*J*,*K*) nor *ex vivo* florescence imaging of spinal cords (Fig. 4*H*,*I*) was sensitive enough to detect the 35% improvement in transplant survival verified by stereology. Based on *post hoc* power analyses, a large number of animals would be required to detect a difference with either of these methods (≥14). This decreases the utility of both *in vivo* bioluminescent imaging and *ex vivo* fluorescent imaging as primary screens for transplant survival in the injured rat spinal cord. In theory, bioluminescent and fluorescent imaging can detect as few as 1000 cells (Terrovitis et al., 2010); in practice, several factors limit light production/detection. For spinal cord transplants, the location of the spinal cord deep within the vertebral column is particularly problematic due to tissue light absorption. Use of longer light wavelengths (i.e., far-red/near-infrared wavelengths) may circumvent this problem. Following SCI, several conditions within the transplant/injury site impact D-luciferin availability (e.g., altered vasculature) or enzymatic activity (e.g., reduced levels of required luciferin co-factors, ATP and O_2_), which substantially impact light production and contribute to the variability in light detected between and within cases. This makes it difficult to ascertain whether different light levels detected arise from variability in the number of cells initially transplanted, delivery of D-luciferin to the cells, or differences in survival. Fluorescence-based probes overcome the limitations associated with D-luciferin administration. Far-red/near-infrared constructs for IVIS imaging have been developed (Shcherbo et al., 2007; Rumyantsev et al., 2016). They may facilitate *in vivo* imaging of cells following transplantation. One concern, however, is our failure to detect differences in transplant survival with *ex vivo* fluorescent imaging of GFP transplants where penetration of light through the tissue (i.e., bone, muscle, skin) is not required. This suggests additional technological advances are required for the use of fluorescence-based assays of transplant survival in whole tissues or animals. Current methods to quantify the number of surviving cells are either time-consuming (e.g., stereology), or require pulverization of the tissue (western blotting, qPCR) which prevents further examination, or either have too much variability or are not sensitive enough to detect anything but robust changes in survival (*in vivo* bioluminescent imaging and *ex vivo* fluorescent imaging). Additional rapid, sensitive approaches for screening transplant survival are needed to facilitate identification of prosurvival strategies. Until better assessments are developed, stereological quantification remains the most reliable method.

### Summary and conclusions

To advance the field, and to maximize the therapeutic use and benefits of cellular transplants for human clinical use, there is a critical need to develop strategies that effectively promote and permit rapid assessment of transplant survival. We have identified the narrow time window in which transplanted cells die within the injured spinal cord, thus establishing the time window in which cytoprotection should be targeted to counteract transplanted cell death. We tested the effects of elevating HIF-1α in cells, and identify HIF-1α as a transcription factor that protects transplanted cells. Lastly, we tested three approaches to quantifying transplant survival, and demonstrate that stereology remains the most reliable until faster, more sensitive methods can be developed. We anticipate that interventions that specifically harness cellular adaptive responses before transplantation could obviate the need to add additional components to cells at the time of transplantation and thus aid in the adoption of this approach to current clinical transplant protocols.

## Synthesis

Reviewing Editor: Jeffery Twiss, University of South Carolina Decisions are customarily a result of the Reviewing Editor and the peer reviewers coming together and discussing their recommendations until a consensus is reached. When revisions are invited, a fact-based synthesis statement explaining their decision and outlining what is needed to prepare a revision will be listed below. The following reviewer(s) agreed to reveal their identity: Patricia Ward, Murray Blackmore.

Manuscript # eN-NWR-0092-19

Title: Hypoxia Inducible Factor 1 alpha (HIF-1α) counteracts the acute death of cells transplanted into the injured spinal cord

Cell transplant is an imminent therapy and a logical extension of the crossroads of organ transplant and advances in cellular engineering. The experiments here show that “priming” a Schwann cell transplant can enhance their survival in vivo. Prior work has established Schwann cell survival to be quite low, as most cells die in the early days after transplantation to the spinal cord. Hif1a offers some protection in other transplantation paradigms. This manuscript substantiates the rapid loss of Schwann Cells, indicates a small benefit to forced expression of Hif1a in this specific paradigm. Transplanted cell numbers can potentially be tracked in vivo by bioluminescence, and alternative imaging techniques (ex vivo fluorescence, in vivo bioluminescence) to monitor cell survival as an alternative to labor-intensive stereology is quite appealing. But the current work seems to provide a negative result regarding bioluminescence as a useful tool as it was deployed here. Overall, it is useful to the field to know that these approaches were tried, as they are perfectly reasonable, and that effects were small. The manuscript is well written, most of the statistical tests are appropriate (minor exceptions below), and most of the experiments seem to be well done. The questions being asked are somewhat disparate, and accordingly the manuscript is a bit fragmented in its current state with none of its three questions definitively answered. However, the reviewers and reviewing editor feel that this manuscript does provide an advance, and the general approach may also be applicable to other cell types. So the we think that this work fits within the scope of and is worthy of further consideration by eNeuro.

Major concerns:

1. Some important technical details are hard to find in the manuscript. These transplants were allografts, not autografts? Were the rats immunosuppressed? If not, how does this impact cell survival? This could be a point of discussion. In addition, it would be very helpful to include a table of animals used in the various experiments, and make very clear how many were analyzed and how many were excluded for various reasons (also see point # 5 below).

2. Approaches to improve the survival of cell transplants are required to move the field towards efficacy and translational benefits not just for SCI but other neurological disorders in which cells are lost. In the context of SCI, targeting the HIF pathway in Schwann cells increased the number of cells surviving the transplant. If this approach results in better functional recovery, there are multiple methods to prime HIF in vitro prior to transplant, so this is translationally appealing. The authors state that the approach utilized in this paper “should be amenable to clinical translation for multiple cell types across the transplantation field”. However, it remains to be tested whether multiple cell types will respond to HIF stabilization in a similar manner. Given the ubiquitous expression of HIF, there are no doubt multiple regulatory processes that are likely cell-type specific (which the authors allude to in lines 600-603). Therefore, it is unknown whether targeting the HIF pathway will have beneficial effects in other cell types. Indeed, in the experiments presented within, HIF1a elevation increased cell death when the unfolded protein response was induced. Thus, the authors should soften statements in the abstract (line 22) as well as in line 71, and specifically note the cell context variability issue in the discussion. Regardless of a powerful priming effect, the need to address cell death pathways will likely not be completely eliminated as the authors have written.

3. The authors convincingly show the presence of HIF1a mRNA expression in Figure 2 and classical HIF target upregulation at the protein level in the cytosolic fraction after VP16-HIF viral transfection. However, the actual protein level of HIF1a in the nuclear fraction is not shown and instead VP16 is used as a surrogate marker. This brings into question the direct causal nature of VEGF and enolase upregulation. A brief explanation of why HIF1a protein was not directly and quantitatively assessed is needed. If there was no detectable HIF1a protein stabilization in either the nuclear or cytosolic fractions, this is also noteworthy and deserves comment. If the western blotting was unsuccessful for some reason then this should be mentioned. If it was performed but failed to show a convincing difference, the authors should consider showing those data.

The RT-PCR data indicate a fairly low level of transgene expression. The effect on target genes is described as “doubling” but from the graph it appears that VEGF increases ~2.2X and enolase by just 1.7X. The exact values should be given, and placed into some context for the reader, meaning how this compares to other approaches. This point of low expression is addressed in the discussion but some mention should be moved to the results, so that the reader understands that the subsequent data probably reflect the impact of a relatively small boost in Hif1a expression.

The authors indicate that constitutively high overexpression of HIF can induce tumor formation and state that the VP16-HIF paradigm they utilized here resulted in low constitutive expression (lines 644-649). This is a very important point, and the manuscript would be strengthened if quantitative data for levels of HIF protein expression relative to controls and VP16 were provided.

The error bars on the western blot quantification are remarkably small, actually undetectable in some cases (unusual in the reviewing editor's experience with immunoblotting). Is this correct? If the errors are indeed that small, the authors should indicate values in the legend.

4. The protective effect of Hif1a against oxidative stress in Figure 3 is described as “robust,” but it is not clear that this description is appropriate. Robust compared to what? It appears to confer about a 20% boost in survival at an intermediate H2O2 concentration. Again, it would be helpful to offer comparatives to help the reader put this effect in context.

5. For Figure 4, stereology was a primary method utilized and provided an important comparison to methods that many experimenters may be tempted to use due to quicker data acquisition and the ability to survive the animals. Please clarify time point that the images in A-F were obtained. As written it is somewhat confusing given the stated intent to compare the results of the two methods. It may not be necessary to repeat the experiment in order to synchronize, but some discussion of how the difference in timing affects the interpretation of in vivo vs. ex vivo imaging vs. cellular imaging is certainly in order.

It is notable that in E, the GFP+ cells appear more dispersed and appear to occupy a larger area within the lesion. Were all cases receiving the VP16-HIF1a SCs dispersed? In other words, does HIF1a overexpression induce the transplanted SCs to migrate more?

The number of animals in each group is unclear - by counting dots it appears to be 7, 5, 7 but this isn't clear. This must be made explicit in the figure legend.

6. The statistical testing in Figure 4G raises some questions. It is described as a post-hoc, 1-tail Fisher's LSD. The 1-tail aspect is a bit questionable, given the potential of Hif1a to drive cell survival in both directions. 1-tail is usually reserved for iron-clad a priori predictions of the direction of an effect, and I'm not sure this qualifies. It is important to avoid bias in the form of selecting tests that yield a preferred result, and the VP16 vs. VP16-Hif1a difference appears to be on the margin at best. Can the author please address what led to the selection of a Fisher's LSD over more standard post-hoc tests (e.g. Sidak's or Tukey's), and whether these other approaches also yield significance? Or if there is an argument to be made for a 1-tail Fisher LSD as the best test, please provide it?

7. Finally, a point of discussion is that no validation of elevated Hif1a expression or target genes was performed in vivo. This is significant because the transplant environment is potentially hypoxic, especially early after transplantation and prior to any vascularization, and thus the control baseline could shift. In other words, we don't actually know the answer to the key question, which is how the Hif1a activity of the transfected cells compares to control in vivo. This should be discussed.

Minor concerns:

1. It would be useful to include a sentence(s) pointing readers to the rationale for transplanting peripheral SCs instead of a different cell type, as some readers will not be familiar with the SC transplant literature.

2. Line 18. Please clarify whether this is 44000 fold increase in radiance or 44000 increase in cell number.

3. Line 72. It may be very counter-intuitive to some readers why you are activating the HIF pathway to enhance cell transplant survival and simultaneously state that hypoxia is a cell death inducer. The lesion site at 7 days post-injury must be a highly hypoxic environment, although this important point is not discussed.

4. Lines 86-87. It is also of note that embryonic stem cells and/or iPSC-derived cells are frequently cultured in hypoxic conditions (~5% O2).

5. Line 102. Please provide the age of the rats utilized for SC isolation.

6. Line 131. The transactivation domain is not adequately described.

7. Line 260. Please provide the qualifying definitions for a “good” vs “bad” transplant. What were the parameters used to exclude the 5 spinal cords from analysis?

8. Line 559. Please define NSPC.

9. Timing of when cell transplant may be most beneficial to survival is not discussed. The lesion site at 7 days post-injury must be a highly hypoxic environment. Have there been any studies to quantify the time course of hypoxia and recovery of vasculature (normoxia)? In other models of SCI (like hemi-transection that may result in a less hypoxic environment), is SC transplant survival better?

10. Could a cover-slip window over the transplanted cord better visualize the emitted light?

## References

[B1] Afshari FT, Kwok JC, White L, Fawcett JW (2010) Schwann cell migration is integrin-dependent and inhibited by astrocyte-produced aggrecan. Glia 58:857–869. 10.1002/glia.20970 20155822

[B2] Ahuja CS, Wilson JR, Nori S, Kotter MRN, Druschel C, Curt A, Fehlings MG (2017) Traumatic spinal cord injury. Nat Rev Dis Primers 3:17018. 10.1038/nrdp.2017.18 28447605

[B3] Aminova LR, Chavez JC, Lee J, Ryu H, Kung A, LaManna JC, Ratan RR (2005) Prosurvival and prodeath effects of hypoxia-inducible factor-1alpha stabilization in a murine hippocampal cell line. J Biol Chem 280:3996–4003. 10.1074/jbc.M409223200 15557337

[B4] Aminova LR, Siddiq A, Ratan RR (2008) Antioxidants, HIF prolyl hydroxylase inhibitors or short interfering RNAs to BNIP3 or PUMA, can prevent prodeath effects of the transcriptional activator, HIF-1alpha, in a mouse hippocampal neuronal line. Antioxid Redox Sign 10:1989–1998. 10.1089/ars.2008.2039 PMC2612757 18774900

[B5] Anderson KD, Guest JD, Dietrich WD, Bartlett Bunge M, Curiel R, Dididze M, Green BA, Khan A, Pearse DD, Saraf-Lavi E, Widerström-Noga E, Wood P, Levi AD (2017) Safety of autologous human Schwann cell transplantation in subacute thoracic spinal cord injury. J Neurotraum 34:2950–2963. 10.1089/neu.2016.4895 28225648

[B6] Araos J, Sleeman JP, Garvalov BK (2018) The role of hypoxic signalling in metastasis: towards translating knowledge of basic biology into novel anti-tumour strategies. Clin Exp Metastas 35:563–599. 10.1007/s10585-018-9930-x 30171389

[B7] Bakshi A, Keck CA, Koshkin VS, LeBold DG, Siman R, Snyder EY, McIntosh TK (2005) Caspase-mediated cell death predominates following engraftment of neural progenitor cells into traumatically injured rat brain. Brain Res 1065:8–19. 10.1016/j.brainres.2005.09.059 16309635

[B8] Barakat DJ, Gaglani SM, Neravetla SR, Sanchez AR, Andrade CM, Pressman Y, Puzis R, Garg MS, Bunge MB, Pearse DD (2005) Survival, integration, and axon growth support of glia transplanted into the chronically contused spinal cord. Cell Transplant 14:225–240. 10.3727/000000005783983106 15929557

[B9] Barker RA, Dunnett SB, Faissner A, Fawcett JW (1996) The time course of loss of dopaminergic neurons and the gliotic reaction surrounding grafts of embryonic mesencephalon to the striatum. Exp Neurol 141:79–93. 10.1006/exnr.1996.0141 8797670

[B10] Barriga EH, Maxwell PH, Reyes AE, Mayor R (2013) The hypoxia factor Hif-1α controls neural crest chemotaxis and epithelial to mesenchymal transition. J Cell Biol 201:759–776. 10.1083/jcb.201212100 23712262 PMC3664719

[B11] Benita Y, Kikuchi H, Smith AD, Zhang MQ, Chung DC, Xavier RJ (2009) An integrative genomics approach identifies hypoxia inducible factor-1 (HIF-1)-target genes that form the core response to hypoxia. Nucleic Acids Res 37:4587–4602. 10.1093/nar/gkp425 19491311 PMC2724271

[B12] Cerqueira SR, Lee YS, Cornelison RC, Mertz MW, Wachs RA, Schmidt CE, Bunge MB (2018) Decellularized peripheral nerve supports Schwann cell transplants and axon growth following spinal cord injury. Biomaterials 177:176–185. 10.1016/j.biomaterials.2018.05.049 29929081 PMC6034707

[B13] Chandel NS, McClintock DS, Feliciano CE, Wood TM, Melendez JA, Rodriguez AM, Schumacker PT (2000) Reactive oxygen species generated at mitochondrial complex III stabilize hypoxia-inducible factor-1alpha during hypoxia: a mechanism of O2 sensing. J Biol Chem 275:25130–25138. 10.1074/jbc.M001914200 10833514

[B14] Chen W, Ostrowski RP, Obenaus A, Zhang JH (2009) Prodeath or prosurvival: two facets of hypoxia inducible factor-1 in perinatal brain injury. Exp Neurol 216:7–15. 10.1016/j.expneurol.2008.10.016 19041643 PMC2672430

[B15] Chen X, Zhang X, Larson CS, Baker MS, Kaufman DB (2006) In vivo bioluminescence imaging of transplanted islets and early detection of graft rejection. Transplantation 81:1421–1427. 10.1097/01.tp.0000206109.71181.bf 16732180

[B16] Chi JT, Wang Z, Nuyten DS, Rodriguez EH, Schaner ME, Salim A, Wang Y, Kristensen GB, Helland A, Børresen-Dale AL, Giaccia A, Longaker MT, Hastie T, Yang GP, van de Vijver MJ, Brown PO (2006) Gene expression programs in response to hypoxia: cell type specificity and prognostic significance in human cancers. PLoS Med 3:e47. 10.1371/journal.pmed.0030047 16417408 PMC1334226

[B17] Chu K, Jung KH, Kim SJ, Lee ST, Kim J, Park HK, Song EC, Kim SU, Kim M, Lee SK, Roh JK (2008) Transplantation of human neural stem cells protect against ischemia in a preventive mode via hypoxia-inducible factor-1alpha stabilization in the host brain. Brain Res 1207:182–192. 10.1016/j.brainres.2008.02.043 18371939

[B18] Dengler VL, Galbraith M, Espinosa JM (2014) Transcriptional regulation by hypoxia inducible factors. Crit Rev Biochem Mol 49:1–15. 10.3109/10409238.2013.838205 24099156 PMC4342852

[B19] Ding WX, Ni HM, Gao W, Hou YF, Melan MA, Chen X, Stolz DB, Shao ZM, Yin XM (2007) Differential effects of endoplasmic reticulum stress-induced autophagy on cell survival. J Biol Chem 282:4702–4710. 10.1074/jbc.M609267200 17135238

[B20] Emgård M, Hallin U, Karlsson J, Bahr BA, Brundin P, Blomgren K (2003) Both apoptosis and necrosis occur early after intracerebral grafting of ventral mesencephalic tissue: a role for protease activation. J Neurochem 86:1223–1232. 10.1046/j.1471-4159.2003.01931.x 12911630

[B21] Forsythe JA, Jiang BH, Iyer NV, Agani F, Leung SW, Koos RD, Semenza GL (1996) Activation of vascular endothelial growth factor gene transcription by hypoxia-inducible factor 1. Mol Cell Biol 16:4604–4613. 10.1128/mcb.16.9.4604 8756616 PMC231459

[B22] Golden KL, Pearse DD, Blits B, Garg MS, Oudega M, Wood PM, Bunge MB (2007) Transduced Schwann cells promote axon growth and myelination after spinal cord injury. Exp Neurol 207:203–217. 10.1016/j.expneurol.2007.06.023 17719577 PMC3513343

[B23] Gomez-Sanchez JA, Carty L, Iruarrizaga-Lejarreta M, Palomo-Irigoyen M, Varela-Rey M, Griffith M, Hantke J, Macias-Camara N, Azkargorta M, Aurrekoetxea I, De Juan VG, Jefferies HB, Aspichueta P, Elortza F, Aransay AM, Martínez-Chantar ML, Baas F, Mato JM, Mirsky R, Woodhoo A, et al. (2015) Schwann cell autophagy, myelinophagy, initiates myelin clearance from injured nerves. J Cell Biol 210:153–168. 10.1083/jcb.201503019 26150392 PMC4494002

[B24] Halterman MW, Federoff HJ (1999) HIF-1alpha and p53 promote hypoxia-induced delayed neuronal death in models of CNS ischemia. Exp Neurol 159:65–72. 10.1006/exnr.1999.7160 10486175

[B25] Halterman MW, Miller CC, Federoff HJ (1999) Hypoxia-inducible factor-1alpha mediates hypoxia-induced delayed neuronal death that involves p53. J Neurosci 19:6818–6824. 10.1523/JNEUROSCI.19-16-06818.1999 10436039 PMC6782875

[B26] Hill CE, Moon LD, Wood PM, Bunge MB (2006) Labeled Schwann cell transplantation: cell loss, host Schwann cell replacement, and strategies to enhance survival. Glia 53:338–343. 10.1002/glia.20287 16267833

[B27] Hill CE, Hurtado A, Blits B, Bahr BA, Wood PM, Bartlett BM, Oudega M (2007) Early necrosis and apoptosis of Schwann cells transplanted into the injured rat spinal cord. Eur J Neurosci 26:1433–1445. 10.1111/j.1460-9568.2007.05771.x 17880386

[B28] Hill CE, Guller Y, Raffa SJ, Hurtado A, Bunge MB (2010) A calpain inhibitor enhances the survival of schwann cells in vitro and after transplantation into the injured spinal cord. J Neurotraum 27:1685–1695. 10.1089/neu.2010.1272 20568964 PMC2966856

[B29] Iwai H, Nori S, Nishimura S, Yasuda A, Takano M, Tsuji O, Fujiyoshi K, Toyama Y, Okano H, Nakamura M (2014) Transplantation of neural stem/progenitor cells at different locations in mice with spinal cord injury. Cell Transplant 23:1451–1464. 10.3727/096368913X670967 23998989

[B30] Karimi-Abdolrezaee S, Eftekharpour E, Wang J, Morshead CM, Fehlings MG (2006) Delayed transplantation of adult neural precursor cells promotes remyelination and functional neurological recovery after spinal cord injury. J Neurosci 26:3377–3389. 10.1523/JNEUROSCI.4184-05.2006 16571744 PMC6673854

[B31] Karlsson J, Emgård M, Brundin P (2002) Comparison between survival of lazaroid-treated embryonic nigral neurons in cell suspensions, cultures and transplants. Brain Res 955:268–280. 10.1016/s0006-8993(02)03601-6 12419547

[B32] Kim DE, Tsuji K, Kim YR, Mueller FJ, Eom HS, Snyder EY, Lo EH, Weissleder R, Schellingerhout D (2006) Neural stem cell transplant survival in brains of mice: assessing the effect of immunity and ischemia by using real-time bioluminescent imaging. Radiology 241:822–830. 10.1148/radiol.2413050466 17114629

[B33] Kumagai G, Okada Y, Yamane J, Nagoshi N, Kitamura K, Mukaino M, Tsuji O, Fujiyoshi K, Katoh H, Okada S, Shibata S, Matsuzaki Y, Toh S, Toyama Y, Nakamura M, Okano H (2009) Roles of ES cell-derived gliogenic neural stem/progenitor cells in functional recovery after spinal cord injury. PLoS One 4:e7706. 10.1371/journal.pone.0007706 19893739 PMC2768792

[B34] Kung AL, Wang S, Klco JM, Kaelin WG, Livingston DM (2000) Suppression of tumor growth through disruption of hypoxia-inducible transcription. Nat Med 6:1335–1340. 10.1038/82146 11100117

[B35] Lendahl U, Lee KL, Yang H, Poellinger L (2009) Generating specificity and diversity in the transcriptional response to hypoxia. Nat Rev Genet 10:821–832. 10.1038/nrg2665 19884889

[B37] Lu P, Wang Y, Graham L, McHale K, Gao M, Wu D, Brock J, Blesch A, Rosenzweig ES, Havton LA, Zheng B, Conner JM, Marsala M, Tuszynski MH (2012) Long-distance growth and connectivity of neural stem cells after severe spinal cord injury. Cell 150:1264–1273. 10.1016/j.cell.2012.08.020 22980985 PMC3445432

[B38] McDonald JW, Liu XZ, Qu Y, Liu S, Mickey SK, Turetsky D, Gottlieb DI, Choi DW (1999) Transplanted embryonic stem cells survive, differentiate and promote recovery in injured rat spinal cord. Nat Med 5:1410–1412. 10.1038/70986 10581084

[B39] Morrissey TK, Kleitman N, Bunge RP (1991) Isolation and functional characterization of Schwann cells derived from adult peripheral nerve. J Neurosci 11:2433–2442. 1869923 10.1523/JNEUROSCI.11-08-02433.1991 PMC6575499

[B40] Mundt-Petersen U, Petersen A, Emgård M, Dunnett SB, Brundin P (2000) Caspase inhibition increases embryonic striatal graft survival. Exp Neurol 164:112–120. 10.1006/exnr.2000.7407 10877921

[B41] Murry CE, Jennings RB, Reimer KA (1986) Preconditioning with ischemia: a delay of lethal cell injury in ischemic myocardium. Circulation 74:1124–1136. 10.1161/01.cir.74.5.1124 3769170

[B42] Nakao N, Frodl EM, Duan WM, Widner H, Brundin P (1994) Lazaroids improve the survival of grafted rat embryonic dopamine neurons. Proc Natl Acad Sci USA 91:12408–12412. 10.1073/pnas.91.26.12408 7809050 PMC45447

[B43] Ney PA (2015) Mitochondrial autophagy: origins, significance, and role of BNIP3 and NIX. Biochim Biophys Acta 1853:2775–2783. 10.1016/j.bbamcr.2015.02.022 25753537

[B44] Nishimura S, Yasuda A, Iwai H, Takano M, Kobayashi Y, Nori S, Tsuji O, Fujiyoshi K, Ebise H, Toyama Y, Okano H, Nakamura M (2013) Time-dependent changes in the microenvironment of injured spinal cord affects the therapeutic potential of neural stem cell transplantation for spinal cord injury. Mol Brain 6:3. 10.1186/1756-6606-6-3 23298657 PMC3556141

[B45] Okada S, Ishii K, Yamane J, Iwanami A, Ikegami T, Katoh H, Iwamoto Y, Nakamura M, Miyoshi H, Okano HJ, Contag CH, Toyama Y, Okano H (2005) In vivo imaging of engrafted neural stem cells: its application in evaluating the optimal timing of transplantation for spinal cord injury. FASEB J 19:1839–1841. 10.1096/fj.05-4082fje 16141363

[B46] Ozdemir M, Attar A, Kuzu I, Ayten M, Ozgencil E, Bozkurt M, Dalva K, Uckan D, Kılıc E, Sancak T, Kanpolat Y, Beksac M (2012) Stem cell therapy in spinal cord injury: in vivo and postmortem tracking of bone marrow mononuclear or mesenchymal stem cells. Stem Cell Rev 8:953–962. 10.1007/s12015-012-9376-5 22552878

[B47] Patel V, Joseph G, Patel A, Patel S, Bustin D, Mawson D, Tuesta LM, Puentes R, Ghosh M, Pearse DD (2010) Suspension matrices for improved Schwann-cell survival after implantation into the injured rat spinal cord. J Neurotraum 27:789–801. 10.1089/neu.2008.0809 20144012 PMC2943946

[B48] Pearse DD, Sanchez AR, Pereira FC, Andrade CM, Puzis R, Pressman Y, Golden K, Kitay BM, Blits B, Wood PM, Bunge MB (2007) Transplantation of Schwann cells and/or olfactory ensheathing glia into the contused spinal cord: survival, migration, axon association, and functional recovery. Glia 55:976–1000. 10.1002/glia.20490 17526000

[B49] Piltti KM, Salazar DL, Uchida N, Cummings BJ, Anderson AJ (2013) Safety of epicenter versus intact parenchyma as a transplantation site for human neural stem cells for spinal cord injury therapy. Stem Cell Transl Med 2:204–216. 10.5966/sctm.2012-0110 23413374 PMC3659765

[B50] Pugh CW (2016) Modulation of the hypoxic response. Adv Exp Med Biol 903:259–271. 10.1007/978-1-4899-7678-9_18 27343102

[B51] Ratan RR, Siddiq A, Aminova L, Langley B, McConoughey S, Karpisheva K, Lee HH, Carmichael T, Kornblum H, Coppola G, Geschwind DH, Hoke A, Smirnova N, Rink C, Roy S, Sen C, Beattie MS, Hart RP, Grumet M, Sun D, et al. (2008) Small molecule activation of adaptive gene expression: tilorone or its analogs are novel potent activators of hypoxia inducible factor-1 that provide prophylaxis against stroke and spinal cord injury. Ann NY Acad Sci 1147:383–394. 10.1196/annals.1427.033 19076458 PMC2921907

[B52] Rehemtulla A, Stegman LD, Cardozo SJ, Gupta S, Hall DE, Contag CH, Ross BD (2000) Rapid and quantitative assessment of cancer treatment response using in vivo bioluminescence imaging. Neoplasia 2:491–495. 10.1038/sj.neo.7900121 11228541 PMC1508085

[B53] Riva C, Donadieu E, Magnan J, Lavieille JP (2007) Age-related hearing loss in CD/1 mice is associated to ROS formation and HIF target proteins up-regulation in the cochlea. Exp Gerontol 42:327–336. 10.1016/j.exger.2006.10.014 17141999

[B54] Robinson J, Lu P (2017) Optimization of trophic support for neural stem cell grafts in sites of spinal cord injury. Exp Neurol 291:87–97. 10.1016/j.expneurol.2017.02.007 28189728

[B55] Roet KC, Eggers R, Verhaagen J (2012) Noninvasive bioluminescence imaging of olfactory ensheathing glia and schwann cells following transplantation into the lesioned rat spinal cord. Cell Transplant 21:1853–1865. 10.3727/096368911X627471 22449606

[B56] Rumyantsev KA, Turoverov KK, Verkhusha VV (2016) Near-infrared bioluminescent proteins for two-color multimodal imaging. Sci Rep 6:36588. 10.1038/srep36588 27833162 PMC5105121

[B57] Ruthenborg RJ, Ban JJ, Wazir A, Takeda N, Kim JW (2014) Regulation of wound healing and fibrosis by hypoxia and hypoxia-inducible factor-1. Mol Cells 37:637–643. 10.14348/molcells.2014.0150 24957212 PMC4179131

[B58] Ryan HE, Lo J, Johnson RS (1998) HIF-1 alpha is required for solid tumor formation and embryonic vascularization. EMBO J 17:3005–3015. 10.1093/emboj/17.11.3005 9606183 PMC1170640

[B59] Schödel J, Mole DR, Ratcliffe PJ (2013) Pan-genomic binding of hypoxia-inducible transcription factors. Biol Chem 394:507–517. 10.1515/hsz-2012-0351 23324384

[B60] Semenza GL (2007) Hypoxia-inducible factor 1 (HIF-1) pathway. Sci STKE 2007:cm8. 10.1126/stke.4072007cm8 17925579

[B61] Semenza GL (2011) Hypoxia-inducible factor 1: regulator of mitochondrial metabolism and mediator of ischemic preconditioning. Biochim Biophys Acta 1813:1263–1268. 10.1016/j.bbamcr.2010.08.006 20732359 PMC3010308

[B62] Semenza GL (2012) Hypoxia-inducible factors in physiology and medicine. Cell 148:399–408. 10.1016/j.cell.2012.01.021 22304911 PMC3437543

[B63] Semenza GL, Roth PH, Fang HM, Wang GL (1994) Transcriptional regulation of genes encoding glycolytic enzymes by hypoxia-inducible factor 1. J Biol Chem 269:23757–23763. 8089148

[B64] Semenza GL, Jiang BH, Leung SW, Passantino R, Concordet JP, Maire P, Giallongo A (1996) Hypoxia response elements in the aldolase A, enolase 1, and lactate dehydrogenase A gene promoters contain essential binding sites for hypoxia-inducible factor 1. J Biol Chem 271:32529–32537. 10.1074/jbc.271.51.32529 8955077

[B65] Shcherbo D, Merzlyak EM, Chepurnykh TV, Fradkov AF, Ermakova GV, Solovieva EA, Lukyanov KA, Bogdanova EA, Zaraisky AG, Lukyanov S, Chudakov DM (2007) Bright far-red fluorescent protein for whole-body imaging. Nat Methods 4:741–746. 10.1038/nmeth1083 17721542

[B66] Shin JC, Kim KN, Yoo J, Kim IS, Yun S, Lee H, Jung K, Hwang K, Kim M, Lee IS, Shin JE, Park KI (2015) Clinical trial of human fetal brain-derived neural stem/progenitor cell transplantation in patients with traumatic cervical spinal cord injury. Neural Plast 2015:630932. 10.1155/2015/630932 26568892 PMC4619963

[B67] Smirnova NA, Rakhman I, Moroz N, Basso M, Payappilly J, Kazakov S, Hernandez-Guzman F, Gaisina IN, Kozikowski AP, Ratan RR, Gazaryan IG (2010) Utilization of an in vivo reporter for high throughput identification of branched small molecule regulators of hypoxic adaptation. Chem Biol 17:380–391. 10.1016/j.chembiol.2010.03.008 20416509 PMC4327942

[B68] Stokes RA, Cheng K, Deters N, Lau SM, Hawthorne WJ, O'Connell PJ, Stolp J, Grey S, Loudovaris T, Kay TW, Thomas HE, Gonzalez FJ, Gunton JE (2013) Hypoxia-inducible factor-1α (HIF-1α) potentiates β-cell survival after islet transplantation of human and mouse islets. Cell Transplant 22:253–266. 10.3727/096368912X647180 22710383 PMC6595221

[B69] Tabakow P, Jarmundowicz W, Czapiga B, Fortuna W, Miedzybrodzki R, Czyz M, Huber J, Szarek D, Okurowski S, Szewczyk P, Gorski A, Raisman G (2013) Transplantation of autologous olfactory ensheathing cells in complete human spinal cord injury. Cell Transplant 22:1591–1612. 24007776 10.3727/096368912X663532

[B70] Takahashi Y, Tsuji O, Kumagai G, Hara CM, Okano HJ, Miyawaki A, Toyama Y, Okano H, Nakamura M (2011) Comparative study of methods for administering neural stem/progenitor cells to treat spinal cord injury in mice. Cell Transplant 20:727–739. 10.3727/096368910X536554 21054930

[B71] Terrovitis JV, Smith RR, Marbán E (2010) Assessment and optimization of cell engraftment after transplantation into the heart. Circ Res 106:479–494. 10.1161/CIRCRESAHA.109.208991 20167944 PMC2826722

[B72] Tetzlaff W, Okon EB, Karimi-Abdolrezaee S, Hill CE, Sparling JS, Plemel JR, Plunet WT, Tsai EC, Baptiste D, Smithson LJ, Kawaja MD, Fehlings MG, Kwon BK (2011) A systematic review of cellular transplantation therapies for spinal cord injury. J Neurotraum 28:1611–1682. 10.1089/neu.2009.1177 20146557 PMC3143488

[B73] Theus MH, Wei L, Cui L, Francis K, Hu X, Keogh C, Yu SP (2008) In vitro hypoxic preconditioning of embryonic stem cells as a strategy of promoting cell survival and functional benefits after transplantation into the ischemic rat brain. Exp Neurol 210:656–670. 10.1016/j.expneurol.2007.12.020 18279854

[B74] Thomas LW, Ashcroft M (2019) Exploring the molecular interface between hypoxia-inducible factor signalling and mitochondria. Cell Mol Life Sci 76:1759–1777. 10.1007/s00018-019-03039-y 30767037 PMC6453877

[B75] Vangeison G, Carr D, Federoff HJ, Rempe DA (2008) The good, the bad, and the cell type-specific roles of hypoxia inducible factor-1 alpha in neurons and astrocytes. J Neurosci 28:1988–1993. 10.1523/JNEUROSCI.5323-07.2008 18287515 PMC6671445

[B76] Wakai T, Narasimhan P, Sakata H, Wang E, Yoshioka H, Kinouchi H, Chan PH (2016) Hypoxic preconditioning enhances neural stem cell transplantation therapy after intracerebral hemorrhage in mice. J Cerebr Blood F Met 36:2134–2145. 10.1177/0271678X15613798 PMC5363661 26661220

[B77] Wang W, Huang X, Lin W, Qiu Y, He Y, Yu J, Xi Y, Ye X (2018) Hypoxic preconditioned bone mesenchymal stem cells ameliorate spinal cord injury in rats via improved survival and migration. Int J Mol Med 42:2538–2550. 10.3892/ijmm.2018.3810 30106084 PMC6192716

[B78] Wu W, Chen X, Hu C, Li J, Yu Z, Cai W (2010) Transplantation of neural stem cells expressing hypoxia-inducible factor-1alpha (HIF-1alpha) improves behavioral recovery in a rat stroke model. J Clin Neurosci 17:92–95. 10.1016/j.jocn.2009.03.039 19913430

[B79] Yang C, Jiang L, Zhang H, Shimoda LA, DeBerardinis RJ, Semenza GL (2014) Analysis of hypoxia-induced metabolic reprogramming. Methods Enzymol 542:425–455. 10.1016/B978-0-12-416618-9.00022-4 24862279

[B80] Yu SP, Wei Z, Wei L (2013) Preconditioning strategy in stem cell transplantation therapy. Transl Stroke Res 4:76–88. 10.1007/s12975-012-0251-0 23914259 PMC3728897

[B81] Zhang M, Methot D, Poppa V, Fujio Y, Walsh K, Murry CE (2001) Cardiomyocyte grafting for cardiac repair: graft cell death and anti-death strategies. J Mol Cell Cardiol 33:907–921. 10.1006/jmcc.2001.1367 11343414

